# Equine Hoof Progenitor Cells Display Increased Mitochondrial Metabolism and Adaptive Potential to a Highly Pro-Inflammatory Microenvironment

**DOI:** 10.3390/ijms241411446

**Published:** 2023-07-14

**Authors:** Ariadna Pielok, Martyna Kępska, Zofia Steczkiewicz, Sylwia Grobosz, Lynda Bourebaba, Krzysztof Marycz

**Affiliations:** 1 Department of Experimental Biology, Faculty of Biology and Animal Science, Wroclaw University of Environmental and Life Sciences, Norwida 27B, 50-375 Wroclaw, Polandlynda.bourebaba@upwr.edu.pl (L.B.); 2 International Institute of Translational Medicine, Jesionowa 11, Malin, 55-114 Wisznia Mała, Poland

**Keywords:** equine, hoof, progenitor cells, stem cells, laminitis, adipose stem cells, ASC

## Abstract

Medicinal signaling cells (MSC) exhibit distinct molecular signatures and biological abilities, depending on the type of tissue they originate from. Recently, we isolated and described a new population of stem cells residing in the coronary corium, equine hoof progenitor cells (HPCs), which could be a new promising cell pool for the treatment of laminitis. Therefore, this study aimed to compare native populations of HPCs to well-established adipose-derived stem cells (ASCs) in standard culture conditions and in a pro-inflammatory milieu to mimic a laminitis condition. ASCs and HPCs were either cultured in standard conditions or subjected to priming with a cytokines cocktail mixture. The cells were harvested and analyzed for expression of key markers for phenotype, mitochondrial metabolism, oxidative stress, apoptosis, and immunomodulation using RT-qPCR. The morphology and migration were assessed based on fluorescent staining. Microcapillary cytometry analyses were performed to assess the distribution in the cell cycle, mitochondrial membrane potential, and oxidative stress. Native HPCs exhibited a similar morphology to ASCs, but a different phenotype. The HPCs possessed lower migration capacity and distinct distribution across cell cycle phases. Native HPCs were characterized by different mitochondrial dynamics and oxidative stress levels. Under standard culture conditions, HPCs displayed different expression patterns of apoptotic and immunomodulatory markers than ASCs, as well as distinct miRNA expression. Interestingly, after priming with the cytokines cocktail mixture, HPCs exhibited different mitochondrial dynamics than ASCs; however, the apoptosis and immunomodulatory marker expression was similar in both populations. Native ASCs and HPCs exhibited different baseline expressions of markers involved in mitochondrial dynamics, the oxidative stress response, apoptosis and inflammation. When exposed to a pro-inflammatory microenvironment, ASCs and HPCs differed in the expression of mitochondrial condition markers and chosen miRNAs.

## 1. Introduction

The rise of stem cell-based therapy in regenerative medicine is a direct answer to the need for personalized and efficient treatment strategies in various human and animal conditions [1,2]. With the application of various types of stem cells, the main objective of stem cell-based therapy is to enhance the repair of the targeted cells, tissues, or organs by restoring homeostasis and promoting regeneration [2]. Thus far, several stem cell types have been studied and utilized in clinical trials, including induced pluripotent stem cells (IPSCs) and multipotent mesenchymal stem cells (MSCs). Due to a lack of ethical concerns as well as a relatively simple isolation and culture process, MSCs are being widely applied in different stem cell-based therapies in human and veterinary medicine [1,3].

MSCs can be isolated from several different tissue sources, primarily including bone marrow, adipose tissue, placenta and umbilical cord. Various studies have previously identified distinct molecular signatures and biological abilities for each MSC population depending on the type of tissue they originate from. Therefore, the expected outcomes of MSCs-based therapies may strictly depend on their intrinsic properties and defined secretome, and can provide better insights into the selection of appropriate MSC pools in the context of disease-tailored therapies [4].

Importantly, the origin of stem cells and the condition of the donor play a crucial role in their therapeutic potential [5]. It is apparent that the residing stem cell populations are affected in the course of many diseases, and that their impairment contributes to the evolvement of symptoms [6,7,8,9]. Furthermore, the inflammatory microenvironment negatively affects the residing population of stem cells. Since the state of the donor plays such a vital role in the MSC condition, in general, allogenic MSC therapy seems to have more advantages, as it provides biologically fit cells in an “off-shelf” manner [10].

Recently, we isolated and described a new population of stem cells residing in the coronary corium: hoof progenitor cells (HPCs) [11,12]. HPCs are plastic adherent, and their capacity for multilineage differentiation was confirmed based on chondrogenesis and osteogenesis results. The expression of two surface markers: Nestin and CD29, was confirmed in HPCs using flow cytometry. Additionally, HPCs were compared to a well-established MSC subset, ASCs. Based on this comparison, we observed that HPCs were characterized by high gene expression of *Nestin*, *K14*, *K15*, *Vegfa*, *CD200*, *Ang1*, *Oct4*, *Sox2* and *Nanog*. We hypothesized that, similar to other stem cell populations, HPCs might also be affected in the course of inflammation, just as ASCs are affected in the course of EMS, a disorder in which inflammation is one of the crucial components for the development of insulin resistance [13,14]. In the case of ASCs isolated from horses with EMS, lower viability, reduced clonogenic potential, senescent phenotype and increased accumulation of oxidative stress factors were observed [13]. Therefore, further research is imperative to fully understand HPCs’ reaction to the inflammatory environment.

Furthermore, due to the location of HPCs in the coronary corium, we hypothesized that they could play a crucial role in the course of laminitis, which is a severe equine foot disorder defined as an inflammation of the laminae—the structure responsible for the attachment of the hoof capsule to the third phalanx (coffin bone) [15,16]. Most often, the cause of laminitis is endocrinopathic [17,18]. The local cellular and humoral milieu of laminitis is characterized by endothelial cell swelling, leukocyte infiltration, erythrocyte accumulation, and the secretion of proinflammatory cytokines such as Il-6, Il-8, Il1β and matrix metalloproteinases [15,19,20,21]. In recent years, more focus has been directed towards understanding the pathophysiology of EMS and laminitis, especially regarding the ramifications for stem cells residing in both adipose tissue and hoofs.

Importantly, attempts to utilize mesenchymal stem cells in laminitis treatment have been proposed. Angelone et al. [21] used ASCs’ platelet-rich plasma, and such treatment was proven to be effective. Whether HPCs could also be utilized in laminitis therapy is currently unknown. Possibly, due to their unique secretome, the result of such intervention could be favorable. It is apparent that further research into the underlying mechanism of the disease and a search for new therapeutic strategies and targets, including MSCs, is necessary. 

The aim of this study was to compare the native populations of ASCs and HPCs in standard culture conditions and their response to the inflammatory milieu that occurs during EMS and laminitis. As the inflammatory microenvironment negatively affects ASCs, we sought to determine how such conditions will affect HPCs. Therefore, we performed priming using a cytokine cocktail mixture (TNFα + IL1β + IFNγ) on these two distinct cell populations. Cellular morphology and metabolism were assessed using methods such as cytometry, qPCR, Western blot and immunofluorescent staining. The presented results could provide insight into HPC metabolism and their regenerative potential in clinical applications.

## 2. Results

### 2.1. ASCs and HPCs Have Similar Morphologies but Exhibit Different Phenotypes

**Figure 1:**
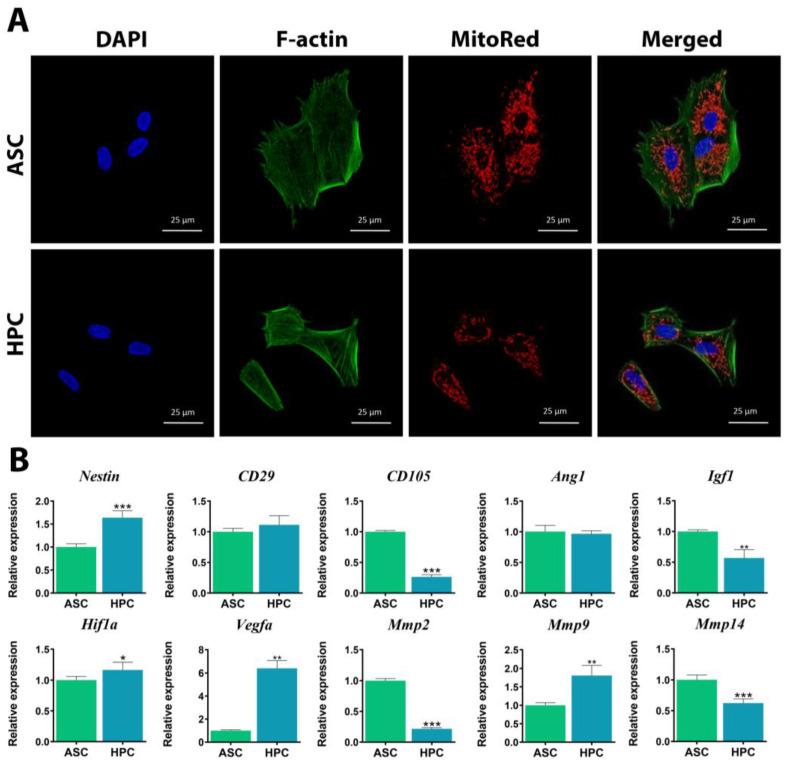
Characterization of ASC and HPC morphology and gene expression of surface markers. (**A**) Representative confocal photographs of ASCs and HPCs labelled with DAPI (blue), phalloidin (green) and MitoRed (red) showcase differences in cellular morphology. (**B**) The phenotypes of ASC and HPC native populations were assessed using RT-qPCR to determine the relative expression analysis of surface markers (*CD105*, *Nestin*, *CD29*), angiogenesis markers (*Ang1*, *Igf1*, *Hif1a*, *Vegfa*) and matrix metalloproteinases (*Mmp2*, *Mmp9*, *Mmp14*). Results are expressed as mean  ±  SD. Statistically significant differences are marked with an asterisk (* *p*  <  0.05, ** *p*  <  0.01, *** *p*  <  0.001).

The general morphology of ASCs and HPCs cells was assessed using confocal microscopy (Figure 1A). Both ASCs and HPCs displayed characteristic, fibroblast-like morphology, with oval-shaped nuclei. In both populations, the cells exhibited a highly developed cytoskeleton with well-defined F-actin tubules and a low nuclear–cytoplasm ratio. The mitochondrial network was evenly distributed in both populations, and the mitochondria appeared in the form of short, separated tubules and round spheres. We sought to identify the differences in the phenotypes of ASC and HPC native populations; therefore, we compared the relative gene expression of surface markers (Figure 1B). The expression of *Nestin* was significantly higher in the HPC population, while the *CD105* transcript was more abundant in the ASC population. There was no difference in the expression of *CD29* between ASCs and HPCs. Furthermore, we observed that ASC and HPC native populations were characterized by disparate expression of angiogenesis markers (Figure 1B). Transcripts such as *Vegfa* (**) and *Hif1a* (*) were more abundant among HPCs, while there was no statistical difference in the expression of *Ang1*, and the expression of *Igf1* (**) was higher in the ASC population. The expression of matrix metalloproteinases (Figure 1B) was significantly higher in the ASC population (Mmp14***, Mmp2***), with the exception of *Mmp9* (**), as the levels of this transcript were higher in HPCs.

### 2.2. HPCs Have Lower Migration Capacity and Different Cell Cycle Dynamics Compared to ASCs

**Figure 2:**
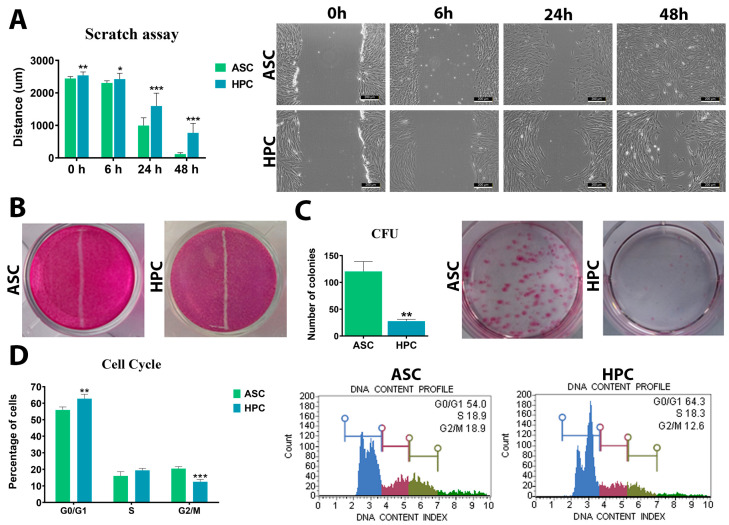
(**A**) Scratch wound healing assay of ASC and HPC native populations. Scale bar = 200 µm. (**B**) Pararosaline staining of the scratch wound healing assay performed 48 h after the scratch wound. (**C**) Analysis of colony-forming efficiency in ASC and HPC native populations. (**D**) The distribution of ASCs and HPCs in the cell cycle (blue-G0/G1; pink-S; olive-G2/M, green-ungated/debris). Results are expressed as mean  ±  SD. Statistically significant differences are marked with an asterisk (* *p*  <  0.05, ** *p*  <  0.01, *** *p*  <  0.001).

The capacity for migration of both native cell populations was assessed using a scratch wound healing assay (Figure 2A,B). The difference between ASCs and HPCs was noticeable at the first time point at 0 h after the scratch wound was performed, with ASCs exhibiting better migration properties. The difference was significant throughout the entire assay, as the wound in the ASC wells was visibly less prominent when compared to HPCs at all time points (Figure 2A,B). Furthermore, native ASCs displayed significantly (**) higher colony-forming efficiency when compared to HPCs (Figure 2C). Differences between ASCs and HPCs were also apparent in the cell cycle dynamic; the HPCs population had a significantly higher percentage of cells in the G0/G1 phase and a lower percentage of cells in the G2/M phase compared to ASCs (Figure 2D).

### 2.3. Native HPCs Possess a Distinct Mitochondrial Metabolism Compared to ASCs

**Figure 3:**
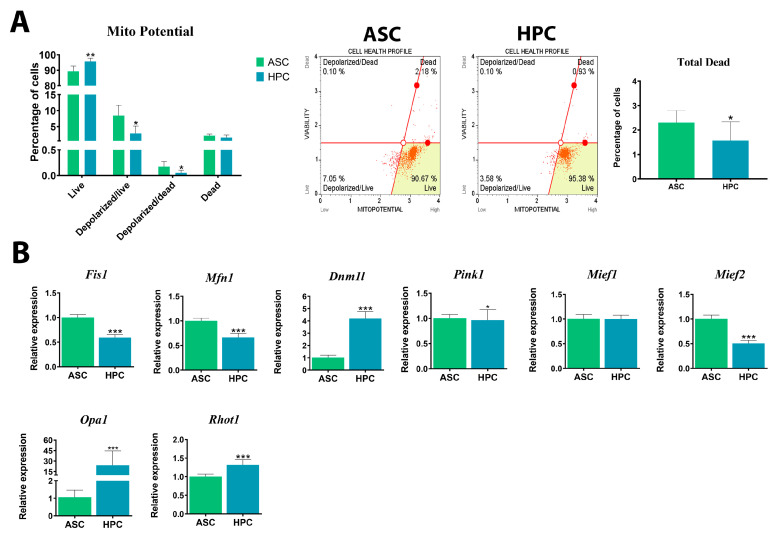
(**A**) Mitochondrial membrane polarization analysis of HPC and ASC native populations. The analysis was performed with the Muse^®^ MitoPotential Kit. (**B**) Gene expression of key mitochondrial dynamics markers (*Fis1*, *Mfn1*, *Dnm1l*, *Pink1*, *Mief2*, *Mief1*, *Opa1*, *Rhot1*). The mitochondrial dynamics in HPC and ASC native populations were tested using a RT-qPCR assay. Results are expressed as mean  ±  SD. Statistically significant differences are marked with an asterisk (* *p*  <  0.05, ** *p*  <  0.01, *** *p*  <  0.001).

The assessment of mitochondrial metabolism in ASC and HPC native populations was based on the analysis of mitochondrial membrane polarization and the gene expression of markers involved in mitochondrial dynamics. The HPC population displayed a significantly (**) higher content of live cells with polarized mitochondrial membranes (Figure 3A). The number of cells with depolarized mitochondrial membranes, whether dead or alive, was higher in the ASC population. Overall, the HPC population was characterized by a lower percentage of dead cells. Furthermore, the gene expression of fission-related *Fis1* (***) and *Mief2* (***) was lower in the HPC population, while the expression of *Dnm1l* was notably higher (Figure 3B). There was no significant difference in the *Mief1* gene expression. At the mRNA level, the expression of *Pink1*, which is involved in mitophagy, was lower in native HPCs, and a similar pattern was observed for the *Mfn1* (***) transcript. Finally, the expression of *Opa1* (**) and *Rhot1* (***) transcripts was significantly higher in HPCs compared to ASCs.

### 2.4. HPCs Exhibit Lower Oxidative Stress Levels Compared to ASCs

**Figure 4:**
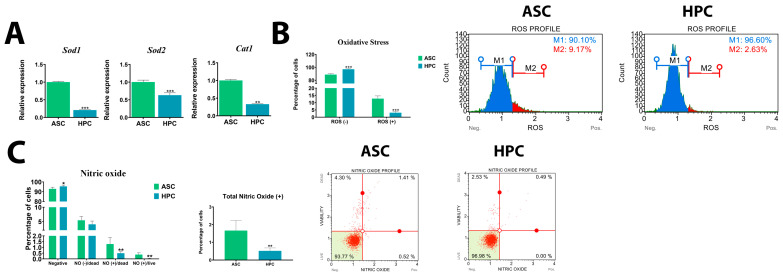
(**A**) Gene expression of key oxidative stress markers (*Sod1*, *Sod2*, *Cat1*) assessed using RT-PCR. (**B**) Characterization of oxidative stress in HPC and ASC native populations. The analysis was performed using the Muse^®^ Oxidative Stress Kit. (**C**) Nitric oxide activity analysis in ASC and HPC native populations. The analysis was performed using the Muse^®^ Nitric Oxide Kit. Results are expressed as mean  ±  SD. Statistically significant differences are marked with an asterisk (* *p*  <  0.05, ** *p*  <  0.01, *** *p*  <  0.001).

The HPC native population exhibited minimal levels of oxidative stress, as evidenced by the reduced percentage of ROS-positive cells (***) (Figure 4B) and a lower expression of key antioxidant enzymes (Sod1***, Sod2***, Cat1**) (Figure 4A). The content of total nitric oxide (**) was also significantly lower in the HPCs (Figure 4C).

### 2.5. HPCs Display Different Gene Expression Patterns of Apoptotic and Immunomodulatory Mediators Compared to ASCs

**Figure 5:**
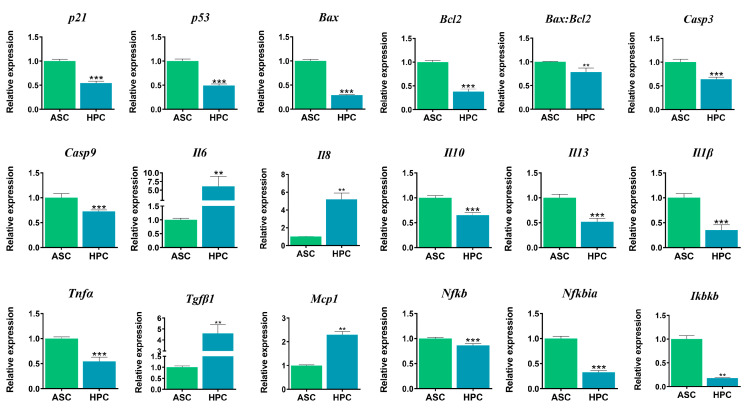
Gene expression of apoptosis markers (*p21*, *p53*, *Bax*, *Bcl2*, *Casp3*, *Casp9*) and immunomodulatory mediators (*Il6*, *Il8*, *Il10*, *Il13*, *Il1b*, *Tnfa*, *Tgfb1*, *Mcp1*, *Nfkb*, *Nfkbia*, *Ikbkb*) in native ASCs and HPCs, assessed using RT-qPCR. Results are expressed as mean  ±  SD.Statistically significant differences are marked with an asterisk (** *p*  <  0.01, *** *p*  <  0.001).

Overall, the expression of key apoptosis markers (Figure 5) was significantly lower in the HPC native population (p21***, p53***, Bax***, Bcl2***, Casp3***, Casp9***), and the *Bax:Bcl2* ratio was also significantly lower (**). The immunomodulatory marker expression varied between the two cell populations. The gene expression of proinflammatory cytokines was significantly higher in the HPCs (Il6**, Il8**); however, the expression of anti-inflammatory cytokines was lower (Il10***, Il13***). Yet, the gene expression of *Tnfα* (***) was higher in the ASC population. Additionally, the expression of *Il1β* (***) was higher in the ASCs. Furthermore, *Tgfβ1* (**) and *Mcp1* (**) mRNA transcripts were more abundant in the HPC population. The expression of genetic markers associated with the *Nfkb* pathway was higher in the ASCs (Ikbkb **, Nfkbia***, Nfkb***).

### 2.6. Priming with a Cytokine Cocktail Exerts a Different Effect on ASC and HPCs’ Mitochondrial Metabolisms

**Figure 6:**
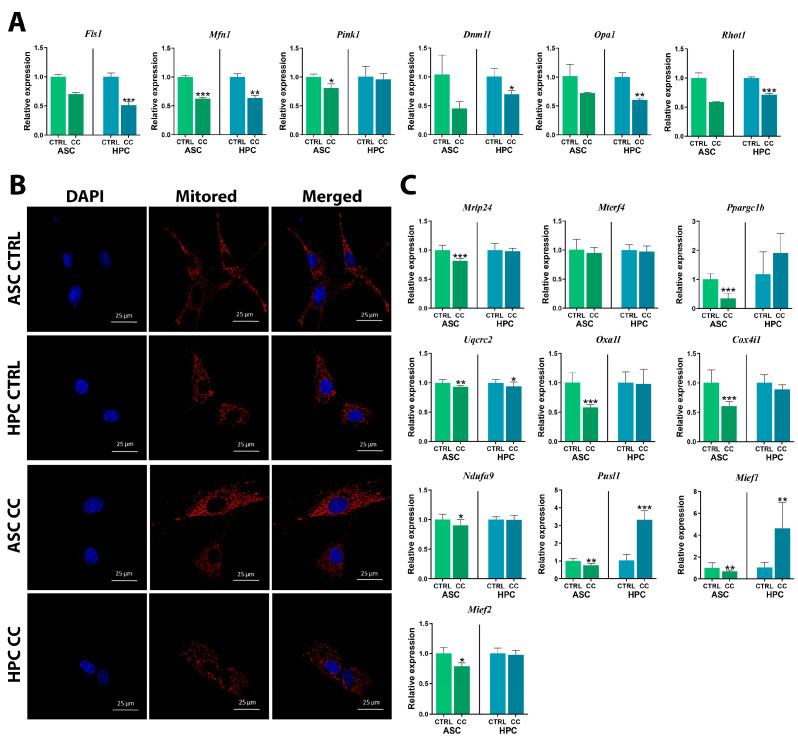
(**A**) Gene expression of key mitochondrial dynamics markers *(Fis1*, *Mfn1*, *Pink1*, *Dnm1l*, *Opa1*, *Rhot1*) in control (CTRL) and cytokine cocktail-treated (CC) ASCs and HPCs, assessed with RT-qPCR. (**B**) Confocal photographs of the control (CTRL) and cytokine cocktail-treated (CC) ASCs and HPCs labelled with DAPI (blue) and MitoRed (red). (**C**) Gene expression of key mitochondrial metabolism markers (*Mrpl24*, *Mterf4*, *Ppargc1b*, *Uqcrc2*, *Oxa1l*, *Coxhi1*, *Ndufa9*, *Pusl1*, *Mief1*, *Mief2*) in control (CTRL) and cytokine cocktail-treated (CC) ASCs and HPCs, assessed using RT-qPCR. Results are expressed as mean  ±  SD. Statistically significant differences are marked with an asterisk (* *p*  <  0.05, ** *p*  <  0.01, *** *p*  <  0.001).

In order to assess the effect of priming on the ASC and HPCs’ mitochondrial networks, microphotographs of untreated cells (CTRL) and cells treated with a TNFα + IL1β + IFNγ (CC) mixture were captured (Figure 6B). Interestingly, for both populations, in the cells subjected to priming, the staining intensity of the mitochondria was higher; however, the difference was more visible in the ASCs (Table S2, Supplementary Materials). Furthermore, we analyzed the gene expression of mitochondrial metabolism markers (Figure 6A,C). Generally, the gene expression of fission, fusion, mitophagy and mitochondrial trafficking-related markers (Figure 6A) either decreased or did not change in both ASCs and HPCs as a result of priming. The expression of *Fis1* and *Mfn1* was significantly downregulated as a result of the cytokine cocktail treatment in HPCs (Fis1***, Mfn1**), while in the ASCs, only *Mfn1* was downregulated (Mfn1***). *Pink1* was downregulated in primed ASCs (*), but no difference in its’ expression was detected in primed HPCs. Both *Dnm1l* (*) and *Opa1* (**) transcripts were less abundant in the HPCs after the incubation with the cytokine cocktail, but there was no statistical difference in the expression of these markers in primed ASCs. *Rhot1* was significantly downregulated in HPCs (***) as a result of priming, but not in ASCs. As for the mitochondrial metabolism markers (Figure 6C), most were downregulated in the primed ASCs, while in HPCs, the gene expression of most markers did not change upon priming. In ASCs treated with the cytokine cocktail mixture, the expression of *Mrlp24*(***) and *Ppargc1b* (***) was significantly downregulated, but there was no significant difference in the expression of *Mterf4*. None of the abovementioned markers showcased any expression changes in the primed HPCs. *Uqcrc2* was significantly downregulated in ASCs (**) and HPCs (*) after incubation in the inflammatory conditions. The expression of *Oxa1l* (***) and *Cox4i1* (***) was only significantly downregulated in primed ASCs. The cytokine cocktail treatment resulted in significant downregulation of *Ndufa9*, but only in ASCs (*), and there was no significant difference in its’ expression in HPCs. *Pusl1* was significantly downregulated in primed ASCs (**), but it was upregulated in primed HPCs (***). *Mief1* expression was significantly downregulated in ASCs (**) after the cytokine cocktail treatment; however, in HPCs (**), it was significantly upregulated. *Mief2* was downregulated in primed ASCs (*), but no change in expression was detected in HPCs. 

### 2.7. Apoptosis Dynamics and Inflammatory Response to Priming Are Similar in HPC and ASC Populations

**Figure 7:**
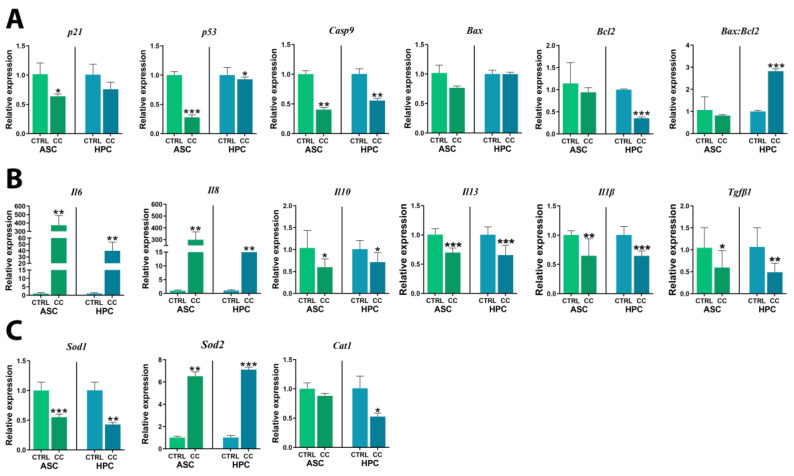
Gene expression of (**A**) apoptosis markers (*p21*, *p53*, *Casp9*, *Bax*, *Bcl2*), (**B**) immunomodulatory mediators (*Il6*, *Il8*, *Il10*, *Il13*, *Il1β*, *Tgfβ*) and (**C**) oxidative stress markers (*Sod1*, *Sod2*, *Cat1*) in control (CTRL) and cytokine cocktail-treated (CC) ASCs and HPCs, assessed with RT-qPCR. Results are expressed as mean  ±  SD. Statistically significant differences are marked with an asterisk (* *p*  <  0.05, ** *p*  <  0.01, *** *p*  <  0.001).

To assess how ASCs and HPCs respond to a highly pro-inflammatory microenvironment, the gene expression of apoptosis, immunomodulatory mediators, and oxidative stress markers was analyzed (Figure 7). Overall, the gene expression of apoptosis markers (Figure 7A) either decreased or did not change in ASCs and HPCs alike. The expression of *p21*(*) and *p53* (***) was significantly lower in ASCs after priming; yet, in HPCs, no significant difference was observed for *p21*, while *p53*(*) was significantly downregulated. *Casp9* expression significantly decreased in ASCs (**) and HPCs (**) after incubation with the cytokine cocktail mixture. There was no difference in the expression of *Bax* in either population, and the *Bcl2* transcript was only significantly less abundant in primed HPCs (***). Furthermore, the *Bax:Bcl2* ratio was significantly higher in HPCs (***) after priming, but no difference was noted for ASCs. Proinflammatory cytokine (Figure 7B) mRNA transcripts were significantly upregulated in ASCs (Il6 **, Il8 **) and HPCs (Il6 **, Il8 **) after the cytokine cocktail treatment. Anti-inflammatory cytokine (Figure 7B) gene expression was downregulated after priming in both ASCs (Il10*, Il13***) and HPCs (Il10*, Il13***). Additionally, *Il1β* and *Tgf β1* were also significantly downregulated in both ASC (Il1β**, Tgf β1*) and HPC (Il1β***, Tgf β1**) primed experimental groups. As for the markers of oxidative stress (Figure 7C), *Sod1* was significantly downregulated in both ASCs (***) and HPCs (**) as a result of priming, contrary to *Sod2*, which was significantly upregulated in both populations (ASCs **, HPCs ***) after the cytokine cocktail treatment. Finally, *Cat1* was significantly downregulated in primed HPCs (*), but no significant difference was noted for the ASCs.

### 2.8. HPC and ASC Native and Primed Populations Display Different miRNA Expression Patterns

**Figure 8:**
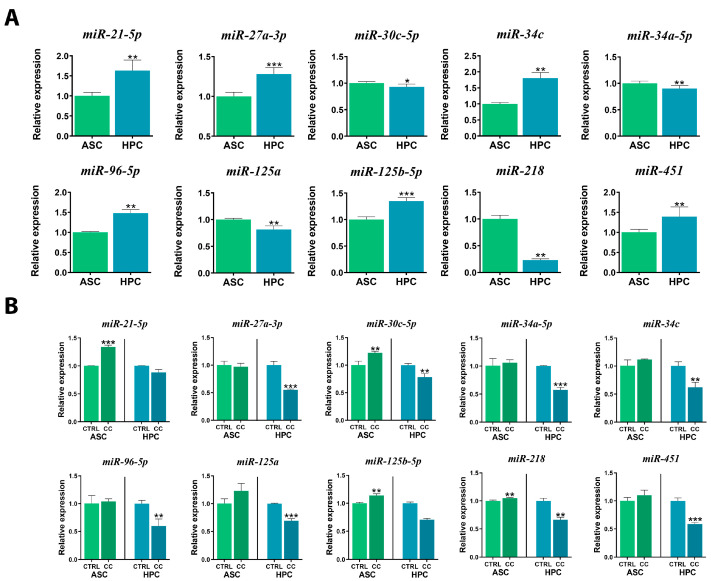
The expression of miRNAs (*miR-21-5p*, *miR-27a*, *miR-30c-5p*, *miR-34a-5p*, *miR34c*, *miR-96-5p*, *miR-125a*, *miR-125b-5p*, *miR-218*, *miR-451*) in (**A**) native ASCs and HPCs, (**B**) cytokine cocktail-treated (CC) ASCs and HPCs, assessed using RT-qPCR. Results are expressed as mean  ±  SD. Statistically significant differences are marked with an asterisk (* *p*  <  0.05, ** *p*  <  0.01, *** *p*  <  0.001).

Due to the crucial regulatory role of miRNAs in various cellular processes, their expression was assessed in HPC and ASC native populations and after priming with the cytokine cocktail mixture (Figure 8A,B). *miR-21-5p* (**), widely known as an onco-miR, was more abundant in the native HPC population, similarly to *miR-27-3p* (***) and *miR-96-5p* (**). The expression of *miR-30c-5p* (*) and *miR-34a-5p* (**) was significantly higher in the native ASCs, while *miR*-*34c* (**) was expressed at a lower level compared to HPCs. Native ASCs exhibited a higher expression of *miR-125a* (**) and *miR-218* (**), and lower levels of *miR-125b-5p* (***) and *miR-451* (**) transcripts.

The expression patterns for miRNAs changed visibly in both populations as a result of priming (Figure 8B). Generally, the expression of miRNAs either did not change or increased after priming in ASCs, while in HPCs, the expression of all studied miRNAs decreased. The expression of *miR-21-5p* was significantly higher (***) in ASCs after priming, while in HPCs, no difference in the expression of the mentioned miRNAs was detected. A similar pattern was observed in *miR-125b-5p* expression (ASCs**). As for *miR-30-5p* (ASCs**, HPCs**) and *miR-218* (ASCs**, HPCs**), their expression was increased in ASCs after priming and decreased in HPCs. Interestingly, there was no significant difference in the expression of *miR-27a-3p*, *miR-34a-5p*, *miR-34c*, *miR-96-5p*, *miR-125a* and *miR-451* in ASCs after incubation with the cytokine cocktail. Yet, in HPCs, all of the above-mentioned markers’ expression was significantly lower after priming. Overall, the expression of all the analyzed miRNAs decreased in HPC cultures after incubation with the cytokine cocktail mixture, and all the differences were statistically significant at (***) for *miR-27a-3p*, (***) for *miR-34a-5p*, (**) for *miR-34c*, (**) *miR-96-5p* and (***) *miR-125a* and *miR-451*(***).

## 3. Discussion

In the past decades, MSC clinical applications have been the focus of numerous studies and clinical trials. The development of stem cell-based therapies is ongoing, and MSCs have been considered for various novel clinical applications, mainly related to their immunomodulatory potential [22,23,24,25]. However, the influence of the environment in which MSCs are expected to exert their immunomodulatory activity plays a huge role in their function [26]. Inflammation is the common link between EMS and laminitis, which strongly modifies the molecular signalling of stromal stem cells [15,19,27,28]. Therefore, it is crucial to establish how inflammatory conditions affect HPCs and ASCs and how this correlates with their clinical potency.

In this study, we investigated the morphology, expansion, apoptosis, and gene expression patterns in ASCs that are commonly used in equine veterinary regenerative medicine practice [29,30], as well as in recently identified HPCs [11,31]. We found that both ASCs and HPCs, although they are of mesenchymal origin, exhibited different expansion, apoptosis, and mitochondrial metabolism, especially when exposed to an in vitro pro-inflammatory microenvironment. 

The morphology of both ASCs and HPCs was similar, consistent with previous observations of mesenchymal cells of various origins isolated from human patients, which typically display a fibroblast-like shape with a prominent nucleus [28,32]. MSC populations are also known to express a panel of cell surface and intracellular markers that define their mesenchymal origin and drive their specific functions. We found that native HPCs displayed higher *Hif1a*, *Vegfa* and *Nestin* transcription capacity when compared to ASCs. *Vegfa* is universally considered to be a marker of angiogenesis and vascular remodelling [33]. However, it has also been reported to partake in energy metabolism regulation [34], especially in adipose tissue. Native HPCs exhibited higher levels of *Vegfa* transcript, which may indicate their potential towards angiogenesis modulation. Numerous studies have addressed the role of angiogenesis and endothelial cells in laminitis [15,35,36]. HPCs expressed higher levels of *Hif1a* transcript, which may suggest their possible innate resistance towards hypoxia. As described by Bingke et al. [37], *Hif1a* can improve MSC survival under hypoxic conditions by improving their viability and inhibiting apoptosis. *Nestin+* MSCs were previously shown to be associated with angiogenesis [38]. Furthermore, expression of *Nestin* was reported as a requirement for the latter astrocytic or neuronal differentiation of MSCs [39]. This could indicate an advantage of HPCs over ASCs in the context of new nerve formation and neuroprotective function. It is especially important in the scope of laminitis, which is associated with neuropathic changes. As Jones et al. reported [40], laminitic horses exhibited abnormal nerve morphology, accompanied by a reduced number of unmyelinated and myelinated fibres. Moreover, both studied populations expressed the *CD105* surface marker, which was interestingly more abundant in the ASC population. However as Maleki et al. [41] demonstrated, depending on the origin, stem cells vary in the expression of endoglin. No difference was observed in the expression of *Ang1* and *CD29*, contrary to our previous results [11]. This discrepancy might be attributed to genetic variation between samples obtained from different animals. HPCs exhibited higher expression of *Mmp9*, but lower expression of *Mmp2* when compared to ASCs. The latter might be associated with HPCs lower migratory capacity [42], as evidenced by the scratch wound healing assay. Gelatinases are associated, among others, with tissue remodeling, angiogenesis [43] and MSC invasion and migration capacity [42]. Furthermore, their involvement in laminitis has also been described [19,44]. *Mmp14* can facilitate the activation of *Mmp2* and *Mmp9*, which are crucial for the degradation of the extracellular matrix and the subsequent migration of MSCs [45]. The higher expression of *Mmp14* in the ASC native population might further suggest their higher capacity for migration. However, lower expression of metalloproteinases involved in connective tissue degradation, which include *Mmp2* and *Mmp14*, potentially indicates that HPCs might exert a beneficial effect on connective tissue regeneration. Although various MSCs have been proposed as good potential candidates for palliating laminitis-induced ECM excessive degradation, other lines of evidence suggest that some stem cells, including ASCs and BM-MSCs, can further participate in proteolytic ECM remodelling as a part of their angiogenic and anti-fibrotic potential. This is accomplished by locally releasing various MMPs such as MMP-2 and MMP-9, which would limit their applicability for laminitis treatment [46]. Here, we found that HPCs express lower levels of MMPs and higher proangiogenic *Vegfa* marker levels, suggesting that HPCs may represent a better alternative in the treatment of laminitis than other MSCs due to their unique paracrine profile. Yet, at the same time, HPCs limited clonogenic potential and expansion in vitro, which might also hinder their long-term clinical applicability and require additional investigation to establish their efficacy using various experimental models and conditions. 

MSC biological and therapeutic properties that include proliferation, migration, expansion and differentiation have been closely associated with organelle dynamics and functions. Thus, mitochondrial biogenesis and metabolism have been highlighted as master maintainers of MSC homeostasis by regulating self-renewal, apoptosis, immunomodulation and multi-directional differentiation, and by collectively determining stem cells fate within the organism [47].

In this study, the observed differential gene expression of selected cell surface markers, angiogenic regulators and matrix turnover regulators might be associated with the distinct mitochondrial dynamics noted in native and primed ASCs and HPCs. The processes of mitochondrial fission and fusion are linked to constant energetic shifts within the cell. While fission is overall related to apoptosis, fusion is associated with its’ inhibition [48,49]. We observed that native HPCs exhibit a dichotomy of fission-related markers, with a lower baseline expression of *Mief2* and *Fis1* and a significantly higher expression of *Dnm1l* compared to ASCs. Notably, such results were previously published in the case of diabetic retinopathy [50] by Zhong and Kowluru, which might be related to the cytosolic location of *Dnm1l*, which is recruited during fission initiation. A similar discrepancy was observed with fusion markers, with *Mfn1* being responsible for inner membrane fusion and *Opa1* being responsible for outer membrane fusion [51]. In general, native ASCs exhibited intensified baseline mitophagy and mitochondria biogenesis compared to HPCs, as evidenced by a higher expression of *Parkin*, *Pgc1a* and *Rhot1* [52,53]. Notably, most mitochondrial fission and fusion-related marker expression decreased in both ASCs and HPCs as a result of the pro-inflammatory environment. Indeed, this deterioration of mitochondrial dynamics has previously been described in ASCs exhibiting impaired immunomodulatory properties that were isolated from patients with type 2 diabetes [54] and older horses [55]. However, HPCs exhibited markedly higher resistance towards inflammation in cases of general mitochondrial condition, metabolism, transcription and mito-ribosomal biogenesis, as evidenced by the unchanged expression of *Mrlp24*, *Oxa1l*, *Cox4il*, *Ndufa9*, *Ppargc1b* and *Mief2* [56,57,58] and the upregulation of *Pusl1* and *Mief1*. Furthermore, the mitophagy dynamics seemed to be retained under a pro-inflammatory milieu in HPCs, indicating their advantage over ASCs in maintaining optimal mitochondrial functions under unfavourable conditions. 

A comparison of the basal immunomodulatory mediator gene expression profiles of both MSCs populations showed that HPCs exhibited a distinct immune reactive molecules transcriptome and were characterized by lower expression of *Il-1β*, *Tnf-α*, *NF-κB* and *Il-10*, as well as higher *Il-6*, *Il-8*, *Mcp-1* and *Tgf-β* transcript levels compared to ASCs. MSC immunomodulatory properties that are essentially mediated by their secretome can be strongly modulated by various extrinsic stimuli, including other tissue sources or the pro-/anti-inflammatory milieu, which further determines the fate of MSCs and their polarization towards either a pro-inflammatory or an immunosuppressive phenotype [59]. Therefore, the ASC and HPC response to pro-inflammatory stimulation was also studied.

Here we have shown that in a high pro-inflammatory milieu, both ASCs and HPCs displayed considerably reduced gene expression levels of the key pro-inflammatory cytokine *Il-1β* and the pro-fibrotic mediator *Tgf-β1*, as well as significant upregulation of *Il-6* and *Il-8* cytokines. Interestingly, the exposure of ASCs and HPCs to the priming cocktail did not enhance the gene expression levels of the anti-inflammatory cytokines *Il-10* and *Il-13*. Several previous studies have examined the impact of MSC preconditioning with various inflammatory cytokines, alone or in combination, and reported differential responses. Wang et al. [60] demonstrated that IFN-γ-primed MCSs from various origins expressed higher levels of immunomodulatory factors including *Ido*, *Pge2*, *Hgf*, *Il-6* and *Il-10*, as well as decreased production of *Ifn-γ* and *Tnf-α*. Sivanathan and colleagues [61] investigated the influence of IL-17 priming and found that treated MSCs presented considerably higher immunosuppressive abilities that were mainly mediated by increased expression of *Il-6*. They concluded that IL-17-stimulated MSCs presented superior immunoregulatory properties over IFN-γ-activated MSCs. Conversely, a functional study showed that TNF-α induction promoted the expression of immunoregulatory factors such as IL-2, PGE2, IDO, and HGF, but with much less intensity compared with IFN-γ priming [62]. As reported by Najar and collaborators, MSCs from different origins can respond differently to cytokines elicitation, and consistent with our model, the exposure of MSCs to a cocktail of cytokines composed of IL-1β, TNF-α, IFN-α and IFN-γ triggered increased synthesis of PGE2 and IL-6. This subsequently mediated the switch of monocyte from the pro-inflammatory M1 phenotype to an anti-inflammatory M2 phenotype [63]. Moreover, the presence of IL-1β in the priming cocktail has been shown to potentiate the transcription of *Cxcr4*, *Cox-2*, *Il-6* and *Il-8* genes, which all participate in the polarization of peritoneal M2 macrophages [64]. In this study, stimulated ASCs and HPCs were characterized by substantially increased *Il-6* and *Il-8* gene expression. This is of particular interest, as both cytokines have been previously reported to play a critical role in boosting the regenerative capacity of MSCs. IL-8 stimulates the release of regenerative signalling molecules that promote MSC survival, angiogenesis and migration through the activation of the PI3K/AKT/MAPK axis [65], while IL-6 has been shown to maintain MSC stemness and stimulate proliferation and wound healing capacity in an ERK1/2-dependent manner [66]. More importantly, the mechanisms driving the MSC phenotype switch are complex and multi-sequential and requires a highly pro-inflammatory milieu. Recently, it has been proposed that under pro-inflammatory conditions, MSCs tend to polarize into an MSC1 phenotype, which enhances the inflammatory response and induces the release of reactive cytokines such as IL-6, IL-8 and GM-CSF. These mediators recruit more neutrophils and macrophages to the inflamed site to further promote inflammation. As a consequence, MSC1 evolves in an environment deprived of anti-inflammatory mediators and is exposed to adequate pro-inflammatory signals, which facilitates the subsequent transition from the MSC1 phenotype to a highly immunosuppressive MSC2 population, promoting tissue repair and regeneration [67]. Therefore, the obtained data is in good agreement with the literature. The increased expression of *Il-6* and *Il-8* and the concomitant *Il-10*, *Il-13* and *Tgf-β* depletion suggest that both ASCs and HPCs may promote the cytokines storm for a more efficient phenotype transition to an immunosuppressive pool of cells with an enhanced migratory and regenerative capacity at early inflammation stages. 

Inflammation is often accompanied by oxidative stress [68,69,70]. In fact, horses with active laminitis have been characterized by increased ROS and RNS generation, excessive lipids peroxidation and overall impaired endogenous antioxidant defences within the injured hoof laminar tissue, causing severe lesions that exacerbate the inflammatory response [71]. Here, we observed that native HPCs displayed lower levels of oxidative stress than ASCs, which correlated with *Sod1*, *Sod2* and *Cat1* expression. However, under inflammatory conditions, both ASCs and HPCs reacted similarly, displaying a protective antioxidant mechanism [72].

Increased apoptosis is another serious laminitis and inflammation hallmark, which is a direct consequence of aberrant cellular lesions and the accumulation of malfunctions. In this study, we observed that native ASCs were characterized by intensified apoptosis compared to HPCs with higher baseline gene expression of proapoptotic markers and a higher *Bax:Bcl2* ratio. In inflammatory conditions, their response was generally similar. However, reduced expression of anti-apoptotic *Bcl2* and an increased *Bax:Bcl2* ratio could indicate more intensive apoptosis in HPCs. Finally, although native HPCs and ASCs exhibited different baseline gene expression patterns of immunomodulatory mediators, their response was similar under inflammatory conditions. Interestingly, at the baseline level, HPCs displayed lower expression of NF-κB signalling pathway markers. Several studies have demonstrated that overexpression of NF-κB and its downstream mediators, IKKs, impairs MSCs stemness and differentiation capacity. Shakibaei and colleagues [73] found that lower activation of the NF-κB pathway increased the production of type II collagen and cartilage-specific proteoglycans during chondrogenesis, further protecting MSCs from apoptosis. Likewise, NF-κB depletion has been shown to potentiate myogenic differentiation [74], while others have reported that NF-κB similarly hampers osteogenic differentiation through β-catenin ubiquitination and Smurf1/Smurf2 activation [75]. The observed lower NF-κB transcripts in HPCs may thus facilitate multilineage differentiation and tissue regeneration. However, additional studies are required to evaluate whether HPCs exhibit higher multipotency compared to other types of MSCs.

In addition to the involvement of cellular organelles and environmental stimuli in MSC behaviour under particular conditions, microRNAs have been largely studied for their role as crucial regulators of stemness, survival, immunomodulation and stem cell differentiation [76,77,78,79,80]. In this context, our data demonstrated a distinct expression pattern of selected miRNAs in native ASCs and HPCs prior to treatment with the cytokine cocktail mixture and after treatment. In the past few decades, a large number of microRNAs have been identified in MSCs from different sources, which have been implicated in the regulation of various cellular processes [81]. For example, Baglio et al. [82] found that human ASCs abundantly secrete *miR-486-5p*, *miR-10a-5p*, *miR-10b-5p*, *miR-191-5p* and *miR-222-3p*. This is in contrast to bone marrow-derived MSCs, in which *miR-143-3p*, *miR-10b-5p*, *miR-486-5p*, *miR-22-3p* and *miR-21-5p* were found at higher levels. Moreover, they reported that *miR-21-5p*, *miR-22-3p*, *miR-10b-5p* and *miR-222-3p* represented the common microRNAs produced by both cell types. Here, we found that equine ASCs only presented higher expression of *miR-218*, *miR-30c-5p* and *miR-125a* over HPCs under basal unstimulated conditions. Interestingly, HPCs displayed higher expression levels of *miR-21-5p*, *miR-27a*, *miR-34c*, *miR-125b-5p*, *miR-451* and *miR-96-5p*, suggesting a distinct microRNA signature in HPCs with a possible impact on cellular biogenesis. To the best of our knowledge, this is the first attempt at profiling equine HPC microRNAs expression, and whether these cells express additional sets of miRNAs, including the *let-7* family members, remains to be determined. However, as described in various investigations, most miRNAs discovered so far, such as *let-7* family subsets, the *miR-23–24-27* clusters, *miR-10*, *miR-29*, *miR-30* and *miR-125*, exert similar effects on various MSCs and modulate their survival, proliferation, stemness, differentiation capacity and immunomodulatory potential. This further suggests that, under physiological conditions, HPCs may present higher cellular flexibility in relation to increased miRNA types and abundances [81]. The MSC microRNA transcriptome profile is also governed by cell milieu and status. In this study, the inflammatory cytokine cocktail strongly affected the HPC miRNome but not ASC miRNome, which seemed to be mostly unaffected. The ASC miRNome reacted with upregulation of only three markers, *miR-21*, *miR-30-5p* and *miR-125b-5p*, which suggest its potential implication in the immunosuppressive mechanisms of ASCs under pro-inflammatory conditions. Decreased levels of virtually all the analyzed miRNAs were observed in HPCs under similar conditions. 

*miR-21* has been described as an important regulator of stem cell differentiation. Furthermore, its expression and secretion via exosomes have been linked to the therapeutic activity MSCs and their immunomodulatory function [83,84,85,86,87]. Notably, even though ASCs exhibited lower baseline expression of *miR-21*, under the inflammatory conditions, the expression increased, while in HPCs, it decreased significantly. Due to the importance of *miR-21* in stem cell regulation, this may suggest an advantage of ASCs, or their tendency towards differentiation in inflammatory environments. It has been previously described that inflammation may mediate signals in the competition between adipocyte and endothelial differentiation of ASC [88]. *miR-30c-5p* loss has been described in mitochondria dysfunction and oxidative stress in hMSCs. Therefore, it is interesting that under inflammatory conditions, HPCs maintained mitophagy, mitoribosome biogenesis and a proper oxidative stress response despite *miR-30c-5p* downregulation [89]. This suggests a possible establishment of miRNA-independent regulation mechanisms in HPCs. Primed HPCs also exhibited lower expression levels of *miR-96*, *miR-125a* and *miR-125b*. Both the *miR-96* and *miR-125* family could be crucial in laminitis treatment. miR-96 has previously been shown to be associated with wound healing, keratinocyte proliferation, migration and the NF-κB signaling pathway [90,91,92,93,94]. The miR-125 family has diverse functions and is involved in neuronal differentiation and injury, as well as self-renewal and differentiation of skin stem cells, among other functions [95,96,97]. *miR-218* which was downregulated in primed HPCs is crucial for MSC osteogenic and chondrogenic differentiation. Furthermore, *miR-218* takes part in regulating skin and hair follicle development [98,99]. Finally, *miR-451* was also downregulated in HPCs under inflammatory conditions. Importantly, its’ immunomodulatory and inflammation-suppressing activity has been described in microglia-mediated neuroinflammation and diabetic retinopathy [100,101]. Collectively, these data indicate a modified HPC microRNAs profile under pro-inflammatory conditions, which questions whether these changes may impact their immunosuppressive and regenerative properties and reduce their clinical potency. Hence, further analysis is required to profile additional microRNAs and perform a comparative study of HPC efficiency under various environmental conditions. The main limitation of this study is the small sample size, as tissue was collected only from three horses. Furthermore, the information regarding age, sex and breed of the horses from the slaughterhouse was limited.

## 4. Materials and Methods

### 4.1. Study Design

**Figure 9:**
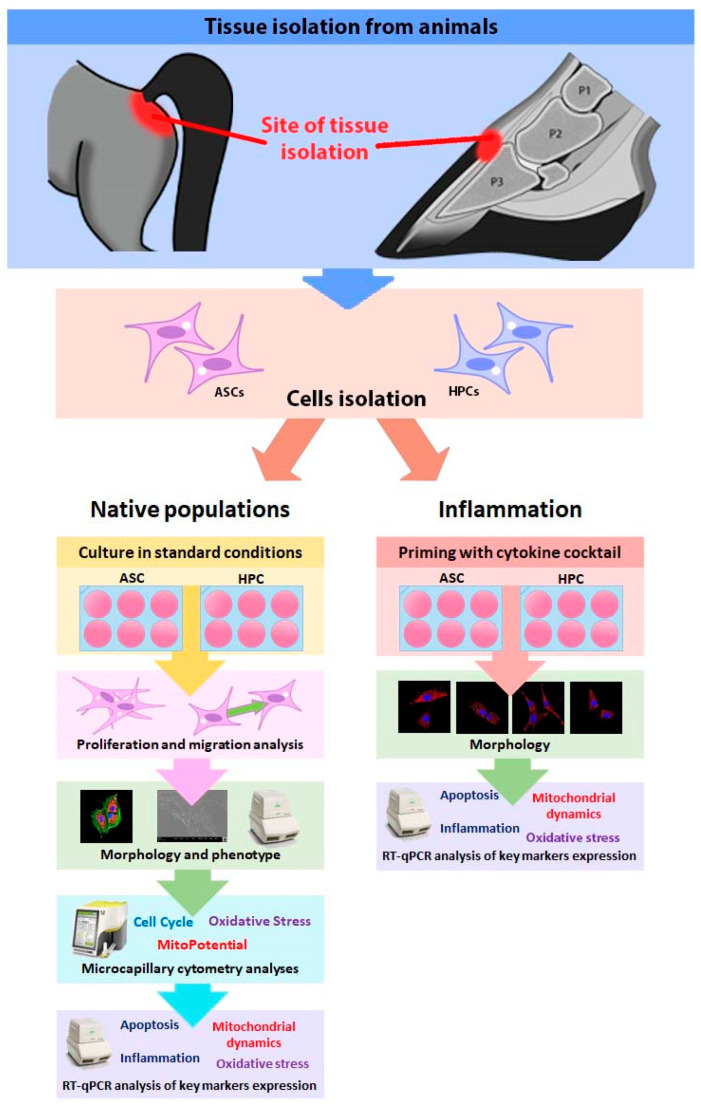
Graphical representation of the study design. P1 (Proximal phalanx bone), P2 (Middle phalanx bone), P3 (Distal phalanx/coffin bone).

Coronary corium tissue samples were collected from 2 young horses, post-mortem, at a local slaughterhouse (Figure 9). Adipose tissue was collected from an 8-year-old warmblood mare from the site surrounding the base of the tail. Following the sample acquisition, ASCs and HPCs were isolated and cultured in both standard conditions (native populations) and in an inflammatory microenvironment created by priming the cells with a cytokine cocktail mixture. The native populations were subjected to immunofluorescent staining in order to visualize their morphology, while their phenotype was assessed based on RT-qPCR expression analysis of chosen surface markers, angiogenesis regulators and matrix metalloproteinases. Furthermore, the migratory capacity of native cells was assessed based on the scratch wound healing assay. The native populations were subjected to a colony forming unit assay in order to determine their clonogenic potential, and their proliferation was assessed based on a cell cycle microcapillary cytometry analysis. Microcapillary cytometry was also used to analyze the mitochondrial membrane potential and oxidative stress in native populations. Additionally, mitochondrial dynamics and oxidative stress in native populations were also validated with RT-qPCR. Autophagy, apoptosis and immunomodulation in the native populations were assessed using RT-qPCR and Western blot techniques. The chosen miRNA expression in native populations was also determined using RT-qPCR. To determine how the inflammatory microenvironment affects HPCs, both ASCs and HPCs were subjected to priming with a cytokine cocktail mixture (TNFα + IL1β + IFNγ) for 18 h. The mitochondrial network in primed ASCs and HPCs was assessed using immunofluorescent staining. Additionally, the mitochondrial dynamics and general mitochondria conditions were determined in primed ASCs and HPCs using RT-qPCR. The expression of key markers for apoptosis, autophagy, oxidative stress and immunomodulation was assessed in primed cells using RT-qPCR. Finally, the expression of the chosen miRNAs was analyzed in primed cells using RT-qPCR.

### 4.2. Ethical Approval

The Local Ethical Committee in Wroclaw for animal experiments approved the study protocol (permit no. 84/2018).

### 4.3. Tissue Harvest and Cell Culture

Samples of coronary corium and adipose tissue were acquired post-mortem from 6 young horses of random sexes, aged between 1–2 years, at the local slaughterhouse. The reasons for the animals’ euthanasia were unrelated to this study. Before the slaughter, the good health and condition of the animals was ensured based on a mandatory clinical examination performed by a veterinarian. The tissue was isolated with a scalpel blade in sterile conditions and subsequently placed in Dulbecco’s modified Eagle’s medium/F12 (DMEM/F12, Sigma Aldrich/Merck, Poznan, Poland) supplemented with a 1% penicillin/streptomycin mix (P/S, Sigma Aldrich, Poznan, Poland). The samples were transported to the lab, and within 1.5 h, HPCs were isolated and cultured according to the protocol described by Yang et al. [12], and ASCs were isolated and cultured as previously described by Marędziak et al. [102]. Briefly, coronary corium and adipose tissue samples were washed 3 times with Hank’s balanced salt solution (HBSS), then minced in a sterile petri dish into 1 × 1 mm squares with a #10 scalpel blade. The minced tissue was transferred into 50 mL sterile falcon tubes containing collagenase type I (1 mg/mL) solution in DMEM/F12 (Sigma Aldrich/Merck, Poznan, Poland) and incubated for 2 h at 37 °C with 2-dimensional agitation for coronary corium, and 40 min at 37 °C for adipose tissue. After digestion, the solution containing coronary corium tissue was filtered, first through a 100 μm filter and then through a 70 μm filter. The solution containing the adipose tissue was not filtered. Next, both samples were centrifuged at 1200× *g* for 10 min, then the supernatant was discarded, and the obtained cell pellet was washed with PBS and centrifuged again at 300× *g* for 4 min. Following the isolation, the obtained HPCs and ASCs from 6 animals were pooled to create one HPC culture and one ASC culture. The HPCs were characterized as previously described [11]. Briefly, at the second passage, the HPCs were analyzed via flow cytometry, which confirmed the expression of Nestin and CD29.The HPCs were cultured in DMEM/F12 (Sigma Aldrich/Merck, Poznan, Poland) supplemented with 10% FBS (fetal bovine serum, heat inactivated, Sigma Aldrich/Merck, Poznan, Poland) and a 1% penicillin/streptomycin mix (P/S, Sigma Aldrich, Poznan, Poland). The ASCs were cultured in Dulbecco’s modified Eagle’s medium (DMEM) containing 1000 mg/L glucose, supplemented with 5% fetal bovine serum (FBS) and a 1% penicillin/streptomycin mix (P/S, Sigma Aldrich, Poznan, Poland). The medium was changed every other day, and the HPCs and ASCs were passaged at 80–90% confluency using a Trypsin/EDTA solution (Gibco Carlsbad, CA, USA). Prior to the experiment, the HPCs and ASCs were cryopreserved in a freezing medium (89% FBS, 10% DMSO, 1% penicillin/streptomycin mix) and thawed before use. Both the ASCs and HPCs used in the experiments were at their 4th passage.

### 4.4. Priming with Cytokine Cocktail

For priming, ASCs and HPCs were incubated with a cytokine cocktail mixture containing TNFα (1000 U/mL), IL1β (50U/mL) and IFNγ (1000 U/mL) for 18 h, then harvested for further analysis.

### 4.5. Cell Morphology Assessment

The morphology of native ASCs and HPCs was assessed using fluorescent staining. For the mitochondrial network visualization, the mitochondria were stained with MitoRed dye (Sigma-Aldrich/Merck, Poznan, Poland) prepared in a culture medium (1:1000). Phalloidin-Atto 488 (Sigma-Aldrich/Merck, Poznan, Poland) was used to visualize the actin cytoskeleton. The nuclei were stained with 4′,6-Diamidine-2′-phenylindole dihydrochloride, and the coverslips were mounted onto glass slides with ProLong™ Diamond Antifade Mountant with DAPI (Thermo Fisher Scientific, Warsaw, Poland). The cells were observed and photographed using a confocal microscope under 630× magnification (Leica TCSSPE, Leica Microsystems, KAWA.SKA Sp. z o. o., Zalesie Górne, Poland). The obtained images were processed using Fiji software (ImageJ 1.52n, Wayne Rasband, National Institute of Health, Bethesda, MD, USA).

### 4.6. Scratch Wound Healing Assay and Colony-Forming Units

The migration of native HPCs and ASCs was assessed using the scratch wound healing assay. The cells were seeded onto a 12-well plate and cultured until fully confluent, then a 10 μL pipette tip was used to create the scratch wound. The cells were then cultured for 48 h, and microphotographs of the wells were taken at 4 time points: 0 h, 6 h, 24 h and 48 h using an inverted Leica DMi1 microscope equipped with a MC170 camera (Leica Microsystems, KAWA.SKA Sp. z o. o., Zalesie Gorne, Poland). After 48 h, the cells were fixed with 4% PFA and stained with a pararosaline solution for the purpose of photographic documentation. The scratch closure was calculated based on the Leica software scale bar (Leica Application Suite- LAS EZ, version 3.4.0), using photographs from 4 wells. To assess the clonogenic potential of native ASCs and HPCs, a colony-forming unit assay was performed as described previously [102,103]. Briefly, 250 cells were seeded onto a 6-well plate and cultured with medium changes every 3 days. After 8 days, the cells were fixed with a 4% PFA solution and stained with a 2% pararosaline solution (Sigma Aldrich/Merck, Poznan, Poland). The number of colonies was counted, and any cluster of 50 cells or more was regarded as a colony.

### 4.7. Microcapillary Cytometry Analyses

ASCs and HPCs were analyzed using commercially available Muse™ reagent kits and the Muse™ Cell Analyzer (Merck, Darmstadt, Germany). Cell cycle analysis was performed using a Muse™ Cell Cycle Assay Kit (Merck, Warsaw, Poland), according to the manufacturers’ protocol. The potential of the mitochondrial membrane in ASCs and HPCs was assessed using the Muse^®^ MitoPotential Assay Kit (Luminex, Austin, TX, USA). A Muse^®^ Oxidative Stress kit (Luminex) was used for the assessment of intracellular oxidative stress factors according to the instructions provided with the kit. In order to detect nitric oxide activity within ASCs and HPCs, a Muse^®^ Nitric Oxide Kit (Luminex) was used in accordance with the manufacturer’s protocol. 

### 4.8. RNA Isolation and qPCR

In order to assess the expression of key markers for apoptosis, immunomodulation, mitochondrial metabolism and miRNAs, the total RNA was isolated from the ASC and HPC cultures using the phenol–chloroform method described previously by Chomczynski et al. [104]. TRIZOL reagent was used according to the instructions provided by the manufacturer. The concentration and purity of the obtained RNA were assessed using a nanospectrophotometer (Epoch, Biotek, Bad Friedrichshall, Germany) at a 260/280 wavelength. A total of 150ng of total RNA was taken for further analysis. The gDNA was digested with RNAse-free DNAse I (Sigma-Aldrich/Merck, Poznan, Poland) and used for cDNA synthesis with a PrimeScript RT Reagent Kit (Takara Bio Europe, Saint-Germaine, Laye, France). For the digestion and synthesis, a T100 Thermal Cycler (Bio-Rad, Hercules, CA, USA) was used according to the manufacturer’s protocol. The obtained cDNA was then diluted with nuclease-free water in a 1:3 ratio and used for the RT-qPCR analysis. The expression of mRNA and miRNA was detected using specific primers (Table S1, Supplementary Materials) and a SensiFAST SYBR and Fluorescein Kit (Meridian Bioscience, London, UK). All the reactions were performed using a CFX Connect™ Real-Time PCR Detection System (Bio-Rad). The exact cycling conditions were previously described [17]. The results obtained from the RT-qPCR analysis were normalized to glyceraldehyde 3-phosphate dehydrogenase (GAPDH) expression for mRNA and U6 for miRNA. The relative expression of each marker was calculated using the 2−ΔΔCQ method [18]. For the native populations, ASCs were used as a reference sample for the calculations, while for the primed cells, the unprimed controls were used as a reference sample.

### 4.9. Mitochondria and mtRNA Isolation

To further examine the status of mitochondria in ASC and HPC cultures in standard and inflammatory conditions, intact mitochondria were isolated from both populations. The isolation was performed using a mitochondria isolation kit for cultured cells (ThermoFisher Scientific, Warsaw, Poland) accordingly to the manufacturer’s protocol. Subsequently, mtRNA was isolated from the extracted mitochondria using the phenol–chloroform method with a TRIZOL reagent, as described above.

### 4.10. Statistical Analysis

At least two biological and three technical replicates of each experiment were performed, and the obtained results are presented as a means ± SD. The normality was assessed using either the Kolmogorov–Smirnov or Shapiro–Wilk test, and the variance was tested with the Fisher test. The statistical differences between the experimental groups were calculated using unpaired Student’s *t*-test or the Mann–Whitney U test. The obtained data were analyzed with GraphPad Prism 8 software (La Jolla, CA, USA). Differences with a probability of *p* < 0.05 were considered significant, and the statistical significance was indicated with an asterisk (*). Furthermore, differences with a probability of *p* < 0.01 were marked with two asterisks (**), and differences with a probability of *p* < 0.001 were marked with three asterisks (***).

## 5. Conclusions

Overall, our findings demonstrated that native HPCs display a similar morphology to ASCs, but possess a distinct phenotype with higher expression levels of *Nestin* stemness-related marker and a lower migration capacity, indicating that HPCs may present a reduced ability to migrate across injured tissues and could potentially exert their effects at a local level rather than systemically. Moreover, HPCs typically express lower levels of MMPs, conferring them better control of defective EMC remodelling. In standard culture conditions, native ASCs and HPCs exhibit different baseline expression of markers commonly involved in mitochondrial dynamics, mitophagy, oxidative stress response, apoptosis and inflammation, whereas HPCs were characterized by higher expression levels of genes involved in mitochondrial metabolism modulation and a higher capacity to express antioxidant enzymes. This suggests a more active cellular machinery and potentially higher metabolic capacities. Importantly, native HPCs and ASCs showcased dissimilar microRNAs expression patterns, and increased expression of *miR-21-5p*, *miR-27-3p*, *miR-34c* and *miR-125b-5p* was observed in HPCs compared to the ASCs population, evoking the ability of HPCs to produce a wide range of reactive microRNAs that are able to modulate various cellular processes. However, under physiological conditions, pro-inflammatory signals induced a drop in the expression of the mentioned miRNAs, which indicates that they may not regulate HPC immunomodulatory properties. Importantly, HPCs appeared to exhibit a distinct immune phenotype under basal and pro-inflammatory conditions compared to ASCs and seemed to specifically express high levels of *Il-6*, *Il-8* and *Tgf-β*. These markers are involved in CD4+ T cell modulation, highlighting the probable implication of HPCs in the regulation of CD4+ T cell fate in response to inflammation. The obtained results do not unequivocally indicate which population might be more suitable for clinical use, as both have certain advantages. However, our results indicated that HPCs may implement different molecular mechanisms for the regulation of inflammatory responses, and could potentially be considered as more suitable for the management of laminitis due to their lower ability to release MMPs, which could worsen ECM degradation. Based on our obtained data, we hypothesize that the combination of ASCs and HPCs or their EVs could be utilized in clinical applications such as laminitis treatment. However, this hypothesis should be further confirmed with ex vivo studies. Additionally, further research is necessary to fully elucidate how the HPC secretome and mitochondrial dynamics might affect their clinical potency. 

## Data Availability

The data presented in this study are available from the corresponding author upon request.

## Supplementary Materials

The following supporting information can be downloaded at: https://www.mdpi.com/article/10.3390/ijms241411446/s1.

## Abbreviations

*Ang1*	Angiopoietin 1
ASCs	Adipose-derived stem cells
*Bax*	BCL-2-associated X protein
BCA	Bicinchoninic acid
*Bcl2*	B cell lymphoma 2
*Casp3*	Caspase 3
*Casp9*	Caspase 9
*Cat1*	Catalase 1
CC	Cytokine cocktail
*Ccl2*	C-C Motif Chemokine Ligand 2
*CD105*	Endoglin
*CD29*	Integrin subunit beta 1
CFU	Colony-forming units
*Cox4il*	Cytochrome c oxidase subunit IV
CTRL	Control
DAMPs	Damage-associated molecular patterns
DMEM/F12	Dulbecco’s modified Eagle’s medium/F12
*Dnm1l*	Dynamin-related protein 1
EAE	Experimental autoimmune encephalomyelitis
EMS	Equine metabolic syndrome
EVs	Extracellular vesicles
*Fis1*	Mitochondrial fission molecule
GAPDH	Glyceraldehyde 3-phosphate dehydrogenase
*Hif1a*	Hypoxia-inducible factor 1 subunit alpha
hMSC	Human mesenchymal stem cells
HPCs	Hoof progenitor cells
HRP	Horseradish peroxidase
IFNγ	Interferon gamma
*Igf1*	Insulin-like growth factor 1
*Ikbkb*	Inhibitor of nuclear factor kappa B kinase subunit beta
*Il6*	Interleukin 6
*Il8*	Interleukin 8
*Il10*	Interleukin 10
*Il13*	Interleukin 13
IL1β	Interleukin 1 beta
IPSCs	Induced pluripotent stem cells
*Mcp1*	Monocyte chemotactic protein 1
*Mfn1*	Mitofusin 1
*Mief1*	Mitochondrial elongation factor 1
*Mief2*	Mitochondrial elongation factor 2
miRNA	microRNA
*Mmp14*	Matrix metallopeptidase 14
*Mmp2*	Matrix metallopeptidase 2
*Mmp9*	Matrix metallopeptidase 9
*Mrlp24*	Mitochondrial ribosomal protein large 24
mRNA	messenger RNA
MSCs	mesenchymal stem cells
*Mterf4*	Mitochondrial transcription termination factor 4
*Ndufa9*	NADH:ubiquinone oxidoreductase subunit A9
*Nfkb*	Nuclear factor kB
*Nfkbia*	NFKB inhibitor alpha
NO	Nitric oxide
*Opa1*	OPA1 mitochondrial dynamin-like GTPase
*Oxa1l*	OXA1L mitochondrial inner membrane protein
P/S	Penicillin/streptomycin mix
*p21*	Cyclin-dependent kinase inhibitor 1A
*p53*	Tumor protein P53
PFA	Paraformaldehyde
*Pink1*	PTEN-induced kinase 1
Ppargc1b	PPARG coactivator 1 beta
*Pusl1*	Pseudouridine synthase-like 1
*Rhot1*	Ras homolog family member T1
RNS	Reactive nitrogen species
ROS	Reactive oxygen species
RT-qPCR	Quantitative reverse transcription polymerase chain reaction
*Sod1*	Superoxide dismutase 1
*Sod2*	Superoxide dismutase 2
*Tgfβ1*	Transforming growth factor beta 1
TNFα	Tumor necrosis factor alpha
*Uqcrc2*	Ubiquinol–cytochrome c reductase core protein 2
*Vegfa*	Vascular endothelial growth factor A

## References

[1] Voga M., Adamic N., Vengust M., Majdic G. (2020). Stem Cells in Veterinary Medicine—Current State and Treatment Options. Front. Vet. Sci..

[2] Hoang D.M., Pham P.T., Bach T.Q., Ngo A.T.L., Nguyen Q.T., Phan T.T.K., Nguyen G.H., Le P.T.T., Hoang V.T., Forsyth N.R. (2022). Stem Cell-Based Therapy for Human Diseases. Signal Transduct. Target. Ther..

[3] Feisst V., Meidinger S., Locke M.B. (2015). From Bench to Bedside: Use of Human Adipose-Derived Stem Cells. Stem Cells Cloning.

[4] Guan Y.T., Xie Y., Li D.S., Zhu Y.Y., Zhang X.L., Feng Y.L., Chen Y.P., Xu L.J., Liao P.F., Wang G. (2019). Comparison of Biological Characteristics of Mesenchymal Stem Cells Derived from the Human Umbilical Cord and Decidua Parietalis. Mol. Med. Rep..

[5] Fan X.L., Zhang Y., Li X., Fu Q.L. (2020). Mechanisms Underlying the Protective Effects of Mesenchymal Stem Cell-Based Therapy. Cell. Mol. Life Sci..

[6] Marzano M., Fosso B., Piancone E., Defazio G., Pesole G., De Robertis M. (2021). Stem Cell Impairment at the Host-Microbiota Interface in Colorectal Cancer. Cancers.

[7] Chatre L., Verdonk F., Rocheteau P., Crochemore C., Chrétien F., Ricchetti M. (2017). A Novel Paradigm Links Mitochondrial Dysfunction with Muscle Stem Cell Impairment in Sepsis. Biochim. Biophys. Acta-Mol. Basis Dis..

[8] Cipriani P., Guiducci S., Miniati I., Cinelli M., Urbani S., Marrelli A., Dolo V., Pavan A., Saccardi R., Tyndall A. (2007). Impairment of Endothelial Cell Differentiation from Bone Marrow–Derived Mesenchymal Stem Cells: New Insight into the Pathogenesis of Systemic Sclerosis. Arthritis Rheum..

[9] Oliva-Olivera W., Coin-Aragüez L., Lhamyani S., Clemente-Postigo M., Torres J.A., Bernal-Lopez M.R., El Bekay R., Tinahones F.J. (2017). Adipogenic Impairment of Adipose Tissue–Derived Mesenchymal Stem Cells in Subjects with Metabolic Syndrome: Possible Protective Role of FGF2. J. Clin. Endocrinol. Metab..

[10] Durand N., Zubair A.C. (2022). Autologous versus Allogeneic Mesenchymal Stem Cell Therapy: The Pros and Cons. Surgery.

[11] Marycz K., Pielok A., Kornicka-Garbowska K. (2021). Equine Hoof Stem Progenitor Cells (HPC) CD29 + /Nestin + /K15 +—A Novel Dermal/Epidermal Stem Cell Population with a Potential Critical Role for Laminitis Treatment. Stem Cell Rev. Rep..

[12] Yang Q., Pinto V.M.R., Duan W., Paxton E.E., Dessauer J.H., Ryan W., Lopez M.J. (2019). In Vitro Characteristics of Heterogeneous Equine Hoof Progenitor Cell Isolates. Front. Bioeng. Biotechnol..

[13] Marycz K., Weiss C., Śmieszek A., Kornicka K. (2018). Evaluation of Oxidative Stress and Mitophagy during Adipogenic Differentiation of Adipose-Derived Stem Cells Isolated from Equine Metabolic Syndrome (EMS) Horses. Stem Cells Int..

[14] Suagee J.K., Corl B.A., Geor R.J. (2012). A Potential Role for Pro-Inflammatory Cytokines in the Development of Insulin Resistance in Horses. Animals.

[15] Katz L.M., Bailey S.R. (2012). A Review of Recent Advances and Current Hypotheses on the Pathogenesis of Acute Laminitis. Equine Vet. J..

[16] Engiles J.B., Galantino-Homer H.L., Boston R., McDonald D., Dishowitz M., Hankenson K.D. (2015). Osteopathology in the Equine Distal Phalanx Associated with the Development and Progression of Laminitis. Vet. Pathol..

[17] Mularczyk M., Bourebaba N., Marycz K., Bourebaba L. (2022). Astaxanthin Carotenoid Modulates Oxidative Stress in Adipose-Derived Stromal Cells Isolated from Equine Metabolic Syndrome Affected Horses by Targeting Mitochondrial Biogenesis. Biomol..

[18] Suszynska M., Poniewierska-Baran A., Gunjal P., Ratajczak J., Marycz K., Kakar S.S., Kucia M., Ratajczak M.Z. (2014). Expression of the Erythropoietin Receptor by Germline-Derived Cells—Further Support for a Potential Developmental Link between the Germline and Hematopoiesis. J. Ovarian Res..

[19] Loftus J.P., Johnson P.J., Belknap J.K., Pettigrew A., Black S.J. (2009). Leukocyte-Derived and Endogenous Matrix Metalloproteinases in the Lamellae of Horses with Naturally Acquired and Experimentally Induced Laminitis. Vet. Immunol. Immunopathol..

[20] Belknap J.K., Faleiros R., Black S.J., Johnson P.J., Eades S. (2011). The Laminar Leukocyte: From Sepsis to Endocrinopathic Models of Laminitis. J. Equine Vet. Sci..

[21] Angelone M., Conti V., Biacca C., Battaglia B., Pecorari L., Piana F., Gnudi G., Leonardi F., Ramoni R., Basini G. (2017). The Contribution of Adipose Tissue-Derived Mesenchymal Stem Cells and Platelet-Rich Plasma to the Treatment of Chronic Equine Laminitis: A Proof of Concept. Int. J. Mol. Sci..

[22] Metcalfe S.M. (2020). Mesenchymal Stem Cells and Management of COVID-19 Pneumonia. Med. Drug Discov..

[23] Cofano F., Boido M., Monticelli M., Zenga F., Ducati A., Vercelli A., Garbossa D. (2019). Mesenchymal Stem Cells for Spinal Cord Injury: Current Options Limitations and Future of Cell Therapy International Journal of Molecular Sciences. Int. J. Mol. Sci..

[24] Zhang K., Jiang Y., Wang B., Li T., Shang D., Zhang X. (2022). Mesenchymal Stem Cell Therapy: A Potential Treatment Targeting Pathological Manifestations of Traumatic Brain Injury. Oxid. Med. Cell. Longev..

[25] Nasiri N., Hosseini S., Reihani-Sabet F., Baghaban Eslaminejad M. (2022). Targeted Mesenchymal Stem Cell Therapy Equipped with a Cell-Tissue Nanomatchmaker Attenuates Osteoarthritis Progression. Sci. Rep..

[26] Boland L., Bitterlich L.M., Hogan A.E., Ankrum J.A., English K. (2022). Translating MSC Therapy in the Age of Obesity. Front. Immunol..

[27] Belknap J.K., Geor R.J. (2016). Equine Laminitis.

[28] Yang Q., Lopez M.J. (2021). The Equine Hoof: Laminitis, Progenitor (Stem) Cells, and Therapy Development. Toxicol. Pathol..

[29] Freitas N.P.P., Silva B.D.P., Bezerra M.R.L., Pescini L.Y.G., Olinda R.G., de Salgueiro C.C.M., Nunes J.F., Martins J.A.M., Martins S.G.N.L.T. (2022). Freeze-Dried Platelet-Rich Plasma and Stem Cell-Conditioned Medium for Therapeutic Use in Horses. J. Equine Vet. Sci..

[30] Golonka P., Kornicka-Garbowska K., Marycz K. (2020). SIRT1+ Adipose Derived Mesenchymal Stromal Stem Cells (ASCs) Suspended in Alginate Hydrogel for the Treatment of Subchondral Bone Cyst in Medial Femoral Condyle in the Horse. Clinical Report. Stem Cell Rev. Rep..

[31] Yang Q. (2019). Equine Hoof Stratum Internum K14+CD105+ Progenitor Cells: Equine Hoof Stratum Internum K14+CD105+ Progenitor Cells: Culture, Characterization, and Model of Epithelial to Mesenchymal Culture, Characterization, and Model of Epithelial to Mesenchymal Transition Transition.

[32] Musina R.A., Bekchanova E.S., Sukhikh G.T. (2005). Comparison of Mesenchymal Stem Cells Obtained from Different Human Tissues. Cell Technol. Biol. Med..

[33] Roy H., Bhardwaj S., Ylä-Herttuala S. (2006). Biology of Vascular Endothelial Growth Factors. FEBS Lett..

[34] Elias I., Franckhauser S., Bosch F. (2013). New Insights into Adipose Tissue VEGF-A Actions in the Control of Obesity and Insulin Resistance. Adipocyte.

[35] Hirschberg R.M., Plendl J. (2005). Pododermal Angiogenesis and Angioadaptation in the Bovine Claw. Microsc. Res. Tech..

[36] Loftus J.P., Black S.J., Pettigrew A., Abrahamsen E.J., Belknap J.K. (2007). Early Laminar Events Involving Endothelial Activation in Horses with Black Walnut– Induced Laminitis. Am. J. Vet. Res..

[37] Lv B., Li F., Fang J., Xu L., Sun C., Han J., Hua T., Zhang Z., Feng Z., Jiang X. (2017). Hypoxia Inducible Factor 1α Promotes Survival of Mesenchymal Stem Cells under Hypoxia. Am. J. Transl. Res..

[38] Xie L., Zeng X., Hu J., Chen Q. (2015). Characterization of Nestin, a Selective Marker for Bone Marrow Derived Mesenchymal Stem Cells. Stem Cells Int..

[39] Wislet-Gendebien S., Wautier F., Leprince P., Rogister B. (2005). Astrocytic and Neuronal Fate of Mesenchymal Stem Cells Expressing Nestin. Brain Res. Bull..

[40] Jones E., Viñuela-Fernandez I., Eager R.A., Delaney A., Anderson H., Patel A., Robertson D.C., Allchorne A., Sirinathsinghji E.C., Milne E.M. (2007). Neuropathic Changes in Equine Laminitis Pain. Pain.

[41] Maleki M., Ghanbarvand F., Behvarz M.R., Ejtemaei M., Ghadirkhomi E. (2014). Comparison of Mesenchymal Stem Cell Markers in Multiple Human Adult Stem Cells. Int. J. Stem Cells.

[42] Ries C., Egea V., Karow M., Kolb H., Jochum M., Neth P. (2007). MMP-2, MT1-MMP, and TIMP-2 Are Essential for the Invasive Capacity of Human Mesenchymal Stem Cells: Differential Regulation by Inflammatory Cytokines. Blood.

[43] Quintero-fabián S., Arreola R., Becerril-villanueva E., Ramírez-camacho M.A., Alvarez-sánchez M.E. (2019). Role of Matrix Metalloproteinases in Angiogenesis and Cancer. Front. Oncol..

[44] Pollitt C.C. (2004). Equine Laminitis: Increased Transcription of Matrix Metalloproteinase-2 (MMP-2) Occurs during the Developmental Phase. Equine Vet. J..

[45] Fu X., Halim A., Tian B., Luo Q., Song G. (2019). MT1-MMP Downregulation via the PI3K/Akt Signaling Pathway Is Required for the Mechanical Stretching-Inhibited Invasion of Bone-Marrow-Derived Mesenchymal Stem Cells. J. Cell. Physiol..

[46] Song Y.H., Shon S.H., Shan M., Stroock A.D., Fischbach C. (2016). Adipose-Derived Stem Cells Increase Angiogenesis through Matrix Metalloproteinase-Dependent Collagen Remodeling. Integr. Biol..

[47] Hsu Y.C., Wu Y.T., Yu T.H., Wei Y.H. (2016). Mitochondria in Mesenchymal Stem Cell Biology and Cell Therapy: From Cellular Differentiation to Mitochondrial Transfer. Semin. Cell Dev. Biol..

[48] Cassidy-Stone A., Chipuk J.E., Ingerman E., Song C., Yoo C., Kuwana T., Kurth M.J., Shaw J.T., Hinshaw J.E., Green D.R. (2008). Chemical Inhibition of the Mitochondrial Division Dynamin Reveals Its Role in Bax/Bak-Dependent Mitochondrial Outer Membrane Permeabilization. Dev. Cell.

[49] Tanaka A., Youle R.J. (2008). A Chemical Inhibitor of DRP1 Uncouples Mitochondrial Fission and Apoptosis. Mol. Cell.

[50] Zhong Q., Kowluru R.A. (2011). Diabetic Retinopathy and Damage to Mitochondrial Structure and Transport Machinery. Investig. Ophthalmol. Vis. Sci..

[51] Li M., Wang L., Wang Y., Zhang S., Zhou G., Lieshout R., Ma B., Liu J., Qu C., Verstegen M.M.A. (2020). Mitochondrial Fusion Via OPA1 and MFN1 Supports Liver Tumor Cell Metabolism and Growth. Cells.

[52] Eiyama A., Okamoto K. (2015). PINK1/Parkin-Mediated Mitophagy in Mammalian Cells. Curr. Opin. Cell Biol..

[53] Safiulina D., Kuum M., Choubey V., Hickey M.A., Kaasik A. (2019). Mitochondrial Transport Proteins RHOT1 and RHOT2 Serve as Docking Sites for PRKN-Mediated Mitophagy. Autophagy.

[54] Marycz K., Alicka M., Major P., Wysocki M. (2019). Adipose-Derived Mesenchymal Stem Cells Isolated from Patients with Type 2 Diabetes Show Reduced “Stemness” through an Altered Secretome Profile, Impaired Anti-Oxidative Protection, and Mitochondrial Dynamics Deterioration. J. Clin. Med..

[55] Alicka M., Kornicka-Garbowska K., Kucharczyk K., Kȩpska M., Rocken M., Marycz K. (2020). Age-Dependent Impairment of Adipose-Derived Stem Cells Isolated from Horses. Stem Cell Res. Ther..

[56] Di Nottia M., Marchese M., Verrigni D., Mutti C.D., Torraco A., Oliva R., Fernandez-Vizarra E., Morani F., Trani G., Rizza T. (2020). A Homozygous MRPL24 Mutation Causes a Complex Movement Disorder and Affects the Mitoribosome Assembly. Neurobiol. Dis..

[57] Bourebaba N., Kornicka-Garbowska K., Marycz K., Bourebaba L., Kowalczuk A. (2021). Laurus Nobilis Ethanolic Extract Attenuates Hyperglycemia and Hyperinsulinemia-Induced Insulin Resistance in HepG2 Cell Line through the Reduction of Oxidative Stress and Improvement of Mitochondrial Biogenesis—Possible Implication in Pharmacotherapy. Mitochondrion.

[58] Yu P., Zhang J., Yu S., Luo Z., Hua F., Yuan L., Zhou Z., Liu Q., Du X., Chen S. (2015). Protective Effect of Sevoflurane Postconditioning against Cardiac Ischemia/Reperfusion Injury via Ameliorating Mitochondrial Impairment, Oxidative Stress and Rescuing Autophagic Clearance. PLoS ONE.

[59] Ragni E., Perucca Orfei C., De Luca P., Mondadori C., Viganò M., Colombini A., De Girolamo L. (2020). Inflammatory Priming Enhances Mesenchymal Stromal Cell Secretome Potential as a Clinical Product for Regenerative Medicine Approaches through Secreted Factors and EV-MiRNAs: The Example of Joint Disease. Stem Cell Res. Ther..

[60] Wang Q., Yang Q., Wang Z., Tong H., Ma L., Zhang Y., Shan F., Meng Y., Yuan Z. (2016). Comparative Analysis of Human Mesenchymal Stem Cells from Fetal-Bone Marrow, Adipose Tissue, and Warton’s Jelly as Sources of Cell Immunomodulatory Therapy. Hum. Vaccines Immunother..

[61] Sivanathan K.N., Rojas-Canales D., Grey S.T., Gronthos S., Coates P.T. (2017). Transcriptome Profiling of IL-17A Preactivated Mesenchymal Stem Cells: A Comparative Study to Unmodified and IFN- γ Modified Mesenchymal Stem Cells. Stem Cells Int..

[62] Prasanna S.J., Gopalakrishnan D., Shankar S.R., Vasandan A.B. (2010). Pro-Inflammatory Cytokines, IFNγ and TNFα, Influence Immune Properties of Human Bone Marrow and Wharton Jelly Mesenchymal Stem Cells Differentially. PLoS ONE.

[63] Miceli V., Bulati M., Iannolo G., Zito G., Gallo A., Conaldi P.G. (2021). Therapeutic Properties of Mesenchymal Stromal/Stem Cells: The Need of Cell Priming for Cell-Free Therapies in Regenerative Medicine. Int. J. Mol. Sci..

[64] Fan H., Zhao G., Liu L., Liu F., Gong W., Liu X., Yang L., Wang J., Hou Y. (2012). Pre-Treatment with IL-1β Enhances the Efficacy of MSC Transplantation in DSS-Induced Colitis. Cell. Mol. Immunol..

[65] Yang A., Lu Y., Xing J., Li Z., Yin X., Dou C., Dong S., Luo F., Xie Z., Hou T. (2018). IL-8 Enhances Therapeutic Effects of BMSCs on Bone Regeneration via CXCR2-Mediated PI3k/Akt Signaling Pathway. Cell. Physiol. Biochem..

[66] Pricola K.L., Kuhn N.Z., Haleem-Smith H., Song Y., Tuan R.S. (2009). Interleukin-6 Maintains Bone Marrow-Derived Mesenchymal Stem Cell Stemness by an ERK1/2-Dependent Mechanism. J. Cell. Biochem..

[67] Ya Loke X., M Imran S.A., Jun Tye G., Safwani Wan Kamarul Zaman W., Nordin F., De Falco E., Pelagalli A., Perteghella S., Kebangsaan Malaysia U., Yaacob Latiff J. (2021). Immunomodulation and Regenerative Capacity of MSCs for Long-COVID. Int. J. Mol. Sci..

[68] Valle-Prieto A., Conget P.A. (2010). Human Mesenchymal Stem Cells Efficiently Manage Oxidative Stress. Stem Cells Dev..

[69] Fukui M., Zhu B.T. (2010). Mitochondrial Superoxide Dismutase SOD2, but Not Cytosolic SOD1, Plays a Critical Role in Protection against Glutamate-Induced Oxidative Stress and Cell Death in HT22 Neuronal Cells. Free Radic. Biol. Med..

[70] Biswas S.K. (2016). Does the Interdependence between Oxidative Stress and Inflammation Explain the Antioxidant Paradox?. Oxid. Med. Cell. Longev..

[71] Laskoski L.M., Dittrich R.L., Valadão C.A.A., Brum J.S., Brandão Y., Brito H.F.V., de Sousa R.S. (2016). Oxidative Stress in Hoof Laminar Tissue of Horses with Lethal Gastrointestinal Diseases. Vet. Immunol. Immunopathol..

[72] Chen K.C., Chen C.R., Chen C.Y., Peng C.C., Peng R.Y. (2022). Bicalutamide Exhibits Potential to Damage Kidney via Destroying Complex i and Affecting Mitochondrial Dynamics. J. Clin. Med..

[73] Buhrmann C., Mobasheri A., Matis U., Shakibaei M. (2010). Curcumin Mediated Suppression of Nuclear Factor-ΚB Promotes Chondrogenic Differentiation of Mesenchymal Stem Cells in a High-Density Co-Culture Microenvironment. Arthritis Res. Ther..

[74] Shakibaei M., Schulze-Tanzil G., John T., Mobasheri A. (2005). Curcumin Protects Human Chondrocytes from IL-1β-Induced Inhibition of Collagen Type II and Β1-Integrin Expression and Activation of Caspase-3: An Immunomorphological Study. Ann. Anat.-Anat. Anz..

[75] Chang J., Liu F., Lee M., Wu B., Ting K., Zara J.N., Soo C., Al Hezaimi K., Zou W., Chen X. (2013). NF-ΚB Inhibits Osteogenic Differentiation of Mesenchymal Stem Cells by Promoting β-Catenin Degradation. Proc. Natl. Acad. Sci. USA.

[76] Collino F., Bruno S., Deregibus M.C., Tetta C., Camussi G. (2011). MicroRNAs and Mesenchymal Stem Cells. Vitam. Horm..

[77] Mei Y., Bian C., Li J., Du Z., Zhou H., Yang Z., Zhao R.C.H. (2013). MiR-21 Modulates the ERK-MAPK Signaling Pathway by Regulating SPRY2 Expression during Human Mesenchymal Stem Cell Differentiation. J. Cell. Biochem..

[78] Trohatou O., Zagoura D., Bitsika V., Pappa K.I., Antsaklis A., Anagnou N.P., Roubelakis M.G. (2014). Sox2 Suppression by MiR-21 Governs Human Mesenchymal Stem Cell Properties. Stem Cells Transl. Med..

[79] Laine S.K., Alm J.J., Virtanen S.P., Aro H.T., Laitala-Leinonen T.K. (2012). MicroRNAs MiR-96, MiR-124, and MiR-199a Regulate Gene Expression in Human Bone Marrow-Derived Mesenchymal Stem Cells. J. Cell. Biochem..

[80] Huang K., Fu J., Zhou W., Li W., Dong S., Yu S., Hu Z., Wang H., Xie Z. (2014). MicroRNA-125b Regulates Osteogenic Differentiation of Mesenchymal Stem Cells by Targeting Cbfβ in Vitro. Biochimie.

[81] Clark E.A., Kalomoiris S., Nolta J.A., Fierro F.A. (2014). Concise Review: MicroRNA Function in Multipotent Mesenchymal Stromal Cells. Stem Cells.

[82] Baglio S.R., Rooijers K., Koppers-Lalic D., Verweij F.J., Pérez Lanzón M., Zini N., Naaijkens B., Perut F., Niessen H.W.M., Baldini N. (2015). Human Bone Marrow- and Adipose-Mesenchymal Stem Cells Secrete Exosomes Enriched in Distinctive MiRNA and TRNA Species. Stem Cell Res. Ther..

[83] Wu Y., Zhang Z., Li J., Zhong H., Yuan R., Deng Z., Wu X. (2022). Mechanism of Adipose-Derived Mesenchymal Stem Cell-Derived Extracellular Vesicles Carrying MiR-21-5p in Hyperoxia-Induced Lung Injury. Stem Cell Rev. Rep..

[84] Wu T., Liu Y., Fan Z., Xu J., Jin L., Gao Z., Wu Z., Hu L., Wang J., Zhang C. (2015). MiR-21 Modulates the Immunoregulatory Function of Bone Marrow Mesenchymal Stem Cells through the PTEN/Akt/TGF-Β1 Pathway. Stem Cells.

[85] Sun Y., Xu L., Huang S., Hou Y., Liu Y., Chan K.M., Pan X.H., Li G. (2015). Mir-21 Overexpressing Mesenchymal Stem Cells Accelerate Fracture Healing in a Rat Closed Femur Fracture Model. Biomed Res. Int..

[86] Wang J., Huang R., Xu Q., Zheng G., Qiu G., Ge M., Shu Q., Xu J. (2020). Mesenchymal Stem Cell-Derived Extracellular Vesicles Alleviate Acute Lung Injury Via Transfer of MiR-27a-3p. Crit. Care Med..

[87] You L., Pan L., Chen L., Gu W., Chen J. (2016). MiR-27a Is Essential for the Shift from Osteogenic Differentiation to Adipogenic Differentiation of Mesenchymal Stem Cells in Postmenopausal Osteoporosis. Cell. Physiol. Biochem..

[88] Ye J., Gimble J.M. (2011). Regulation of Stem Cell Differentiation in Adipose Tissue by Chronic Inflammation. Clin. Exp. Pharmacol. Physiol..

[89] Deun Jung Y., Park S.K., Kang D., Hwang S., Kang M.H., Hong S.W., Moon J.H., Shin J.S., Jin D.H., You D. (2020). Epigenetic Regulation of MiR-29a/MiR-30c/DNMT3A Axis Controls SOD2 and Mitochondrial Oxidative Stress in Human Mesenchymal Stem Cells. Redox Biol..

[90] Huang Y., Zhu N., Chen T., Chen W., Kong J., Zheng W., Ruan J. (2019). Triptolide Suppressed the Microglia Activation to Improve Spinal Cord Injury Through MiR-96/IKKβ/NF-ΚB Pathway. Spine.

[91] Bin Zhan J., Zheng J., Zeng L.Y., Fu Z., Huang Q.J., Wei X., Zeng M. (2021). Downregulation of MiR-96-5p Inhibits MTOR/NF-Κb Signaling Pathway via DEPTOR in Allergic Rhinitis. Int. Arch. Allergy Immunol..

[92] Wu P., Cao Y., Zhao R., Wang Y. (2019). MiR-96-5p Regulates Wound Healing by Targeting BNIP3/FAK Pathway. J. Cell. Biochem..

[93] Uwiera R.R.E., Egyedy A.F., Ametaj B.N. (2017). Laminitis: A Multisystems Veterinary Perspective with Omics Technologies. Periparturient Diseases of Dairy Cows.

[94] Mobasheri A., Critchlow K., Clegg P.D., Carter S.D., Canessa C.M. (2004). Chronic Equine Laminitis Is Characterised by Loss of GLUT1, GLUT4 and ENaC Positive Laminar Keratinocytes. Equine Vet. J..

[95] Zhang L., Stokes N., Polak L., Fuchs E. (2011). Specific MicroRNAs Are Preferentially Expressed by Skin Stem Cells to Balance Self-Renewal and Early Lineage Commitment. Cell Stem Cell.

[96] Boissart C., Nissan X., Giraud-Triboult K., Peschanski M., Benchoua A. (2012). MiR-125 Potentiates Early Neural Specification of Human Embryonic Stem Cells. Development.

[97] Takeda Y.S., Xu Q. (2015). Neuronal Differentiation of Human Mesenchymal Stem Cells Using Exosomes Derived from Differentiating Neuronal Cells. PLoS ONE.

[98] Shi L., Feng L., Liu Y., Duan J.Q., Lin W.P., Zhang J.F., Li G. (2018). MicroRNA-218 Promotes Osteogenic Differentiation of Mesenchymal Stem Cells and Accelerates Bone Fracture Healing. Calcif. Tissue Int..

[99] Chen S., Xu Z., Shao J., Fu P., Wu H. (2019). MicroRNA-218 Promotes Early Chondrogenesis of Mesenchymal Stem Cells and Inhibits Later Chondrocyte Maturation. BMC Biotechnol..

[100] Sun Y., Peng R., Peng H., Liu H., Wen L., Wu T., Yi H., Li A., Zhang Z. (2016). MiR-451 Suppresses the NF-KappaB-Mediated Proinflammatory Molecules Expression through Inhibiting LMP7 in Diabetic Nephropathy. Mol. Cell. Endocrinol..

[101] Sun X., Zhang H. (2018). MiR-451 Elevation Relieves Inflammatory Pain by Suppressing Microglial Activation-Evoked Inflammatory Response via Targeting TLR4. Cell Tissue Res..

[102] Maredziak M., Marycz K., Tomaszewski K.A., Kornicka K., Henry B.M. (2016). The Influence of Aging on the Regenerative Potential of Human Adipose Derived Mesenchymal Stem Cells. Stem Cells Int..

[103] Smieszek A., Marcinkowska K., Pielok A., Sikora M., Valihrach L., Carnevale E., Marycz K. (2022). Obesity Affects the Proliferative Potential of Equine Endometrial Progenitor Cells and Modulates Their Molecular Phenotype Associated with Mitochondrial Metabolism. Cells.

[104] Chomzynski P. (1987). Single-Step Method of RNA Isolation by Acid Guanidinium Thiocyanate–Phenol–Chloroform Extraction. Anal. Biochem..

