# Supplementary material for: Equine Hoof Progenitor Cells Display Increased Mitochondrial Metabolism and Adaptive Potential to a Highly Pro-Inflammatory Microenvironment
Source: Int J Mol Sci. 2023 Jul 14;24(14):11446. doi: 10.3390/ijms241411446 (PMC10379971; doi:10.3390/ijms241411446)
Supplement: Supplementary file 1 [file ijms-24-11446-s001.zip › ijms-2454175-supplementary.pdf]

Table S1. Specific sequences of mRNA and miRNA primers used for RT-qPCR analysis.

Gene		Primers (5'→3')	Length of amplicon	Accession No.
**ANG1**	F:	AGGGTTTCCCAATTAGTCGCT	**137**	XM_001494946.6
	R:	GCTTCGAGCCTGTAGCAGAT		
**BAX**	F:	CGAGTGGCAGCTGAGATGTT	**153**	XM_023650076.1
	R:	AAGGAAGTCCAGTGTCCAGC		
**BCL2**	F:	TTCTTTGAGTTCGGTGGGGT	**164**	XM_001490436.4
	R:	GGGCCGTACAGTTCCACAA		
**CAT1**	F:	ACTCCCATTGCGGTTCGATT	**211**	XM_001914718.5
	R:	TCAGGTGCGTTTGAGGGTTT		
**CASP3**	F:	GGCAGACTTCCTGTATGCGT	**167**	NM_001163961.1
	R:	CCATGGCTACCTTGCGGTTA		
**CASP9**	F:	CACCTTCCCAGGCTTTGTCT	**224**	XM_005607504.3
	R:	GGCTCTGGCCTCAGTAAGTT		
**CD29**	F:	GGCTAACAGGGAGTTTCAGAT	**100**	NM_001301217.1
	R:	ACATCTATTTTCATCTGCTTGGC		
**CD105**	F:	GACTGCCTTTGTGCAGTTGG	**198**	XM_003364144.4
	R:	ATGCTTTCGGGGTCCTTCAG		
**COX4I1**	F:	GAATAGGGGCACGAACGAGT	**138**	XM_023637444.1
	R:	GCCACCCACTCCTCTTCAAA		
**DNM1L**	F:	CAGCTAGTCCACGTTTCACC	**96**	XM_023643342.1
	R:	CCCTTTAGAAAGGTGTCTTGAGT		
**FIS1**	F:	GGTGCGAAGCAAGTACAACG	**118**	XM_001504462.5
	R:	GTTGCCCACAGCCAGATAGA		
**GAPDH**	F:	GATGCCCCAATGTTTGTGA	**250**	NM_001163856.1
	R:	AAGCAGGGATGATGTTCTGG		
**HIF1A**	F:		**183**	XM_023627857.1
	R:	CTCAAATGCAAGAACCTGCTC		

		TTCCATACCATCTTTTGTCACTG		
**IGF1**	**F:**	ATCAGCAGTCTTCCAACCCA	**86**	NM_001082498.2
	**R:**	GAACTGAAGAGCGTCCACCA		
**IKBKB**	**F:**	GGCGGTTGACATTAGCACAG	**395**	XM_023630669.1
	**R:**	CTGAAGCCGAACCACAGTCT		
**IL1B**	**F:**	TATGTGTGTGATGCAGCTGTG	**382**	NM_001082526.1
	**R:**	ACTCAAATTCCACGTTGCCC		
**IL6**	**F:**	CGTCACTCCAGTTGCCTTCT	**225**	NM_001082496.2
	**R:**	GCCAGTACCTCCTTGCTGTT		
**IL8**	**F:**	CTGGCTGTGGCTCTCTTG	**132**	NM_001083951.2
	**R:**	CAGTTTGGGATTGAAAGGTTTG		
**IL10**	**F:**	TGTTGTTGAACGGGTCCCTG	**242**	NM_001082490.1
	**R:**	ACTCTTCACCTGCTCCACTG		
**IL13**	**F:**	AGCTTAGGCCAGCTTACGTG	**179**	XM_023616897.1
	**R:**	TGGGTGATGTTGACCAGCTC		
**K15**	**F:**	AACCAGGAGTACAAGACGCTG	**192**	XM_005597407.3
	**R:**	AGAAACCACCTTTCCGTCCA		
**MCP1**	**F:**	ATTGGCCAAGGAGATCTGTG	**167**	NM_001081931.2
	**R:**	ATATCAGGGGGCATTTAGGG		
**MFN1**	**F:**	AAGTGGCATTTTTCGGCAGG	**217**	XM_005601821.3
	**R:**	TCCATATGAAGGGCATGGGC		
**MIEF1**	**F:**	ATGCTGGGCATCGCTACAC	**284**	XM_023631522.1
	**R:**	CGGAGCCGTGACTTCTTCAA		
**MIEF2**	**F:**	CGTTCTATTATCAGGCAGGTCC	**108**	XM_005597824.3
	**R:**	AGAACTCTGCCATGGTCTTCT		
**MMP2**	**F:**	TCCCACTTTGATGACGACGA	**182**	XM_023637007.1
	**R:**	AAGTTGTAGGTGGTGGAGCA		

**MMP9**	**F:**	TCGTCATCCAGTTTGGCGTT	**145**	NM_001111302.1
	**R:**	TTGCCCAGAGACCACAACTC		
**MMP14**	**F:**	CCTATGCCTACATCCGCGAG	**167**	XM_023621963.1
	**R:**	GGCAGAGTCAAAGTGGGTGT		
**MRPL24**	**F:**	ATGATCCCTAGCGAAGCACC	**123**	XM_001500466.4
	**R:**	TGTAGAGACTCGTACCCGCT		
**MTERF4**	**F:**	CGCCACCTCCGTGCTATG	**73**	XM_001497592.4
	**R:**	CCCAAATGAGGGGCATCAGG		
**NDUFA9**	**F:**	TTGGTATTCAGGCCACACCC	**103**	XM_001494601.4
	**R:**	GCTGGCTTCACGTCTTCAAC		
**Nestin**	**F:**	ACTGAGAAGTTCCAGCTGGC	**158**	XM_023640985.1
	**R:**	TCAGCCTCTAGAAGGGTCC		
**NFKB**	**F:**	CTTCCTTCGAGCCAGTGACG	**84**	XM_001916418.5
	**R:**	CCAGGAGACTTGCTGTCGTG		
**NFKBIA**	**F:**	CACTTCACCTTCGTGAGGCT	**129**	XM_023624022.1
	**R:**	TGTCACAGGACACAACTGGG		
**OPA1**	**F:**	CTTCTCTTGTTAGGTTCACCTG G	**110**	XM_003363363.4
	**R:**	TGTAAGAGAATGAGCTCACC AAG		
**OXA1L**	**F:**	GACCTAGAAACCGTGGGACG	**105**	XM_005603213.3
	**R:**	GGAAGATCACTTGGCTCCCC		
**p21**	**F:**	GAAGAGAAACCCCCAGCTCC	**241**	XM_023633878.1
	**R:**	TGACTGCATCAAACCCCACA		
**p53**	**F:**	TTTCGACATAGCGTGGTGGT	**180**	NM_001202405.1
	**R:**	CTCAAAGCTGTTCCGTCCCA		
**PERK**	**F:**	GTGACTGCAATGGACCAGGA	**283**	XM_023618757.1
	**R:**	TCACGTGCTCACGAGGATATT		
**PINK1**	**F:**	GCACAATGAGCCAGGAGCTA	**298**	XM_014737247.2
	**R:**	GGGGTATTCACGCGAAGGTA		
**PPARGC 1B**	**F:**	CCTCAACTATCTTGCCGACAC C	**165**	XM_023617445.1

	**R:**	ATGGGTTCAGTCTCGGGGT		
**PUSL1**	**F:**<br>**R:**	TCAGCCACTTCCAGGACCTA<br>AGCCACATCCAAGCTGTCTG	**120**	XM_023636049.1
**RHOT1**	**F:**<br>**R:**	GATCCTGCTGGTGGGAGAAC<br>GGGAGGAACCTCTTCTGGGA	**88**	XM_023651633.1
**SOD1**	**F:**<br>**R:**	CATTCCATCATTGGCCGCAC<br>GAGCGATCCCAATCACACCA	**130**	NM_001081826.3
**SOD2**	**F:**<br>**R:**	GGACAAACCTGAGCCCCAAT<br>TTGGACACCAGCCGATACAG	**125**	NM_001082517.2
**SOX2**	**F:**<br>**R:**	ACAGCATGGAGAGAAACCCG<br>GTGGACGGGATTGGTGTTCT	**101**	XM_023623361.1
**TNFA**	**F:**<br>**R:**	AAGTGACAAGCCTGTAGCCC<br>GGTTGACCTTGGACGGGTAG	**254**	NM_001081819.2
**TGFB1**	**F:**<br>**R:**	ATTCCTGGCGCTACCTCAGT<br>GCTGGAACTGAACCCGTTGAT	**197**	NM_001081849.1
**UQCRC2**	**F:**<br>**R:**	TGCTTCGTCTTGCATCCAGT<br>AACTCCGGTGACGTGGTAAC	**193**	XM_001494381.5
**VEGFA**	**F:**<br>**R:**	CCCACTGCGGAGTTCAACAT<br>TTTCTCCGCTCTGAGCAAGG	**167**	NM_001081821.1

**Gene**		**Primer sequence 5′-3′R:**		**Accession no.**
**miR-21**	**Primer sequence 5′-3′R:**	TAGCTTATCAGACTGATGTTGA	**72**	NR_032904.1
**miR-27a**	**Primer sequence 5′-3′R:**	AGGGCUUAGCUGCUUGUGAGC A	**77**	MIMAT0004633
**miR-30c**	**Primer sequence 5′-3′R:**	TGTAAACATCCTACACTCTCAG C	**58**	NR_032885.1
**miR-34a**	**Primer sequence 5′-3′R:**	UGGCAGUGUCUUAGCUGGUUG U	**63**	NR_032803.1

**miR-34c**	**Primer sequence 5′-3′R:**	AGGCAGTGTAGTTAGCTGATTGC	**77**	NR_032871.1
**miR-96**	**Primer sequence 5′-3′R:**	TTTGGCACTAGCACATTTTTGCT	**78**	NR_032832.1
**miR-125a**	**Primer sequence 5′-3′R:**	TCCCTGAGACCCTTTAACCTGTGA	**68**	NR_032888.2
**miR-125b**	**Primer sequence 5′-3′R:**	TCCCTGAGACCCTAACTTGTGA	**70**	NR_033054.1
**miR-218**	**Primer sequence 5′-3′R:**	TAGCTTATCAGACTGATGTTGA	**62**	NR_032813.1
**miR-451**	**Primer sequence 5′-3′R:**	AAACCGTTACCATTACTGTGTT	**72**	NR_032913.1

Table S2. Staining intensity - % of red color pixels measured with Fiji is just ImageJ software – color pixel counter plugin.

Color	Color Pixels	Total Pixels	Particles	% Color	Sample
Red	43262	126025	206009.524	34.328	ASC CTRL
Red	48247	125670	229747.619	38.392	ASC CC
Red	22775	125670	108452.381	18.123	HPC CTRL
Red	23622	126025	112485.714	18.744	HPC CC
